# Selective engineering of condensation properties of single-stranded DNA binding (SSB) protein via its intrinsically disordered linker region

**DOI:** 10.1093/nar/gkaf481

**Published:** 2025-06-06

**Authors:** Péter Ecsédi, Dávid Érfalvy, Zoltán J Kovács, Viktoria Katran, János Pálinkás, Miklós Cervenak, Rita Pancsa, Gábor M Harami, László Smeller, Mihály Kovács

**Affiliations:** ELTE-MTA “Momentum” Motor Enzymology Research Group, De partment of Biochemistry, Eötvös Loránd University, Pázmány P. s. 1/c, H-1117 Budapest, Hungary; ELTE-MTA “Momentum” Motor Enzymology Research Group, De partment of Biochemistry, Eötvös Loránd University, Pázmány P. s. 1/c, H-1117 Budapest, Hungary; ELTE-MTA “Momentum” Motor Enzymology Research Group, De partment of Biochemistry, Eötvös Loránd University, Pázmány P. s. 1/c, H-1117 Budapest, Hungary; HUN-REN–ELTE Motor Pharmacology Research Group, D epartment of Biochemistry, Eötvös Loránd University, Pázmány P. s. 1/c, H-1117 Budapest, Hungary; ELTE-MTA “Momentum” Motor Enzymology Research Group, De partment of Biochemistry, Eötvös Loránd University, Pázmány P. s. 1/c, H-1117 Budapest, Hungary; ELTE-MTA “Momentum” Motor Enzymology Research Group, De partment of Biochemistry, Eötvös Loránd University, Pázmány P. s. 1/c, H-1117 Budapest, Hungary; Department of Biophysics and Radiation Biology, Semmelweis University, Tűzoltó u. 37-47, H-1094 Budapest, Hungary; HUN-REN Institute of Molecular Life Sciences, Research Centre for Natural Sciences, Magyar Tudósok Körútja 2, H-1117 Budapest, Hungary; ELTE-MTA “Momentum” Motor Enzymology Research Group, De partment of Biochemistry, Eötvös Loránd University, Pázmány P. s. 1/c, H-1117 Budapest, Hungary; Department of Biophysics and Radiation Biology, Semmelweis University, Tűzoltó u. 37-47, H-1094 Budapest, Hungary; ELTE-MTA “Momentum” Motor Enzymology Research Group, De partment of Biochemistry, Eötvös Loránd University, Pázmány P. s. 1/c, H-1117 Budapest, Hungary; HUN-REN–ELTE Motor Pharmacology Research Group, D epartment of Biochemistry, Eötvös Loránd University, Pázmány P. s. 1/c, H-1117 Budapest, Hungary

## Abstract

Single-stranded DNA binding (SSB) proteins are essential components of genome metabolism in both bacteria and eukaryotes. Recently demonstrated condensation propensities have placed SSB functions in a new context regarding the organization of nucleic acid-modifying complexes. In this work, we provide functional dissection of the condensation and partner binding properties of *Escherichia coli* (Ec) SSB via engineered modifications of its intrinsically disordered linker (IDL) region. We identify specific alterations in two glycine-rich regions as well as aromatic and/or positively charged residues of the IDL by which a broad-range, selective modification of condensation propensity and condensate thermal and chemical stability can be achieved, while leaving the single-stranded DNA and partner protein binding functions of SSB unchanged. AlphaFold 3-predicted structures of tetrameric wild-type and engineered EcSSB constructs identify multiple possible binding sites for the conserved C-terminal tip on the tetramer core of the IDL, establishing a link between condensation propensity and restrictions in IDL conformational dynamics. Besides defining the contributions of IDL-driven interactions to driving protein condensation, these results pave the way for the definition of *in vivo* roles of EcSSB condensation via genetic engineering and delineate ways for further development of liquid–liquid phase separation prediction algorithms.

## Graphical Abstract

**Graphical Abstract:**
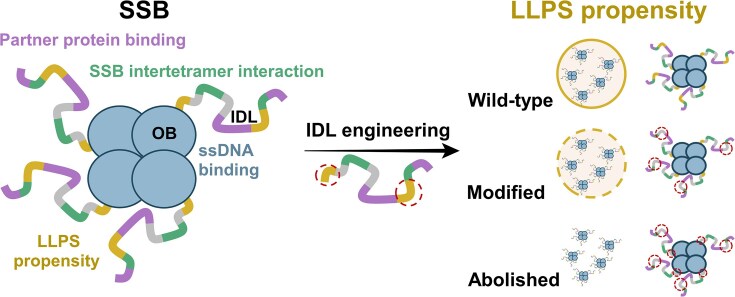

## Introduction

Compartmentalization of the intracellular space is essential as it allows reactions demanding different environments to take place simultaneously, molecules to be stored separately and released only when needed. Eukaryotic cells achieve compartmentalization with membrane-bound organelles and, to a lesser extent, prokaryotes can attain a similar effect with the intrusion of their cell membrane [1]. Besides membrane-based compartmentalization, membraneless organelles can dynamically form via liquid–liquid phase separation (LLPS) [2, 3], which has been shown to be a vitally important organizational principle for a multitude of eukaryotic cellular functions [4, 5]. However, we know of only a handful of phase-separating proteins in prokaryotes [6, 7], albeit the functional role of this mechanism may be especially prominent regarding their limited intracellular space.

Recently, we and others have shown that *Escherichia coli* single-stranded (ss) DNA binding protein (EcSSB), a critically important component of DNA metabolism, forms protein condensates under physiological conditions in an ssDNA-regulated manner, suggesting the general importance of this mechanism in bacterial genome maintenance [8, 9]. It has been proposed that SSB and its partners are stored in cytosolic condensates in the absence of stress and can be rapidly mobilized, driven by exposure of ssDNA segments, upon genome damage [8, 10].

Bacterial SSBs comprise an N-terminal oligonucleotide/oligosaccharide-binding (OB) domain [amino acids (aa) 1–113], responsible for ssDNA binding [11], and an intrinsically disordered linker (IDL, aa 114–178) driving higher-order SSB-nucleoprotein compaction and protein–protein interactions (PPIs) [12, 13]. The C-terminal segment of the IDL (CTP, C-terminal peptide, aa 170–178) is highly conserved among bacteria and is the primary element responsible for PPIs [12, 14, 15–17]. Contributions of these elements to the LLPS propensity of EcSSB have been indicated previously [8, 9]. The OB domains assemble into homotetramers allowing multivalency, a feature generally necessary for LLPS-based condensation. The CTP segments can form intra- and intertetramer interactions with the OB domains, promoting protein association. Nevertheless, SSB variants lacking the CTP can still form protein condensates when assisted by molecular crowders. However, SSB variants lacking the IDL, but retaining the CTP, completely lose their condensation propensity [8, 9].

The N-terminal part of the EcSSB IDL harbors multiple glycine-rich regions (aa 114–120 and aa 124–130), which were suggested to be important for condensation; however, their functional dissection has not been performed [9]. Notably, glycine-rich sequences are key determinants of condensation propensity in other proteins [18, 19]. Furthermore, arginines and aromatic residues in disordered regions can also promote condensation and/or self-assembly in various proteins via cation–pi interactions [20, 21]. The EcSSB IDL harbors several candidates for such interactions [9, 14], but the roles of these residues have not been clarified. Interestingly, the SSB homolog of *Plasmodium falciparum* (PfSSB) is unable to form condensates, and this holds even for a chimeric construct where the IDL of EcSSB is replaced by that of PfSSB (EPE construct, retaining the CTP of EcSSB) [9]. This is presumably due to the low number of glycines and the high number of negatively charged amino acid residues in PfSSB IDL. The human SSB homologs hSSB1 and hSSB2 have also been shown to form condensates, with IDL sequences that are highly divergent and mechanisms that differ from those of EcSSB [22].

In the present work, we sought to dissect the contribution of the structural elements of the IDL to EcSSB condensation and engineer the IDL to create EcSSB variants with reduced or abolished condensation propensity, while retaining the ssDNA-binding and PPI capacities. By analysis of variants harboring changes in the glycine-rich and/or cation–pi interaction-forming elements, we show that fine-tuned IDL–IDL interactions are key drivers of condensation propensity, while CTP exposure can vary independently from condensation. The stability of condensates, as well as partner enrichment in EcSSB condensates, can also be separately engineered in a broad range. Our results identify constructs that will be suitable for the specific determination of the functional contribution of SSB-driven condensation to bacterial physiology.

## Materials and methods

### Cloning, expression, and purification of EcSSB variants and RecQ helicase

Coding sequences for wild-type (WT) EcSSB (UniProt code: P0AGE0) and its variants (described in the “Results” section) were cloned into a modified pET29b vector (His-pET29b, Amp^R^) complemented with the coding sequence for an N-terminal tobacco etch virus (TEV) protease cleavable His_6_-tag. DNA sequences of EcSSB variants were generated by using either conventional oligos [e.g. GG or (GGS)_4_] or megaprimers containing whole IDL sequences (e.g. human or *P. falciparum*) with the coding region of EcSSB CTP.

**Figure 1.**
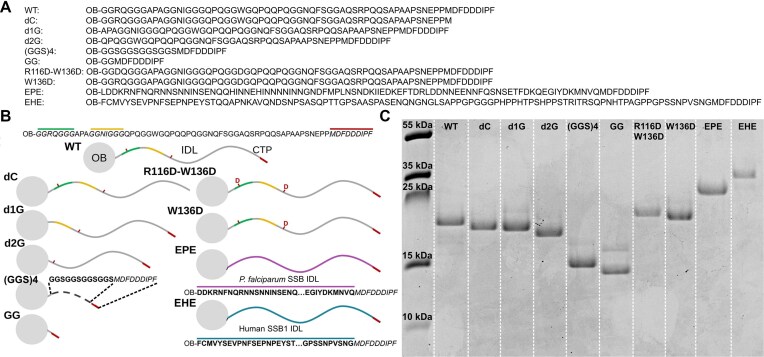
IDL sequences, structure schematics, and SDS–PAGE image of purified EcSSB constructs. (**A**) IDL sequences (starting at aa 114) of EcSSB constructs used in this study. [“OB” represents the OB domain (aa 1–113). The EcSSB CTP sequence is MDFDDDIPF.] (**B**) Structural schematics of EcSSB constructs. Gray sphere and gray line represent the OB domain and the IDL, respectively. CTP is shown as a red segment at the C-terminus of the IDL. Green and yellow sections represent the two G-rich regions of the IDL (green: GGRQGGG; yellow: GGNIGGG). IDLs from *P. falciparum* SSB and human SSB1 are shown in purple and teal, respectively. The (GGS)_4_ linker is presented as a dashed line. Red letters “D” indicate the introduced aspartates (positions 116 and 136, underlined in the WT sequence). (**C**) SDS–PAGE shows that preparations of EcSSB variants are free of WT EcSSB expressed by host bacteria. The lower electrophoretic mobility of the W136D and R116D–W136D constructs is presumably due to more extended IDL conformation resulting from changes in charge patterning.

DNA constructs were transformed into BER2566 cells, which were then grown at 37°C in LB medium containing 100 μg/ml ampicillin. Expression was initiated by adding 1 mM isopropyl β-d-1-thiogalactopyranoside (IPTG) when the opacity of the culture reached an OD_600_ of 0.8. Cells then were shaken at 18°C overnight. On the following day, cells were pelleted (5000 × *g* for 8 min) and then resuspended in a buffer containing 50 mM Tris–HCl, pH 8, 500 mM NaCl, 30 mM imidazole, and 3 mM NaN_3_. Cells were disrupted by ultrasonication and were centrifuged at 48 000 × *g* for 20 min. The supernatants were applied to Ni affinity column. Here, two buffers were used for purification: a wash solution (buffer A: 50 mM Tris–HCl (pH 8), 500 mM NaCl, 30 mM imidazole) and an elution buffer (buffer B: buffer A complemented with 250 mM imidazole). Proteins were eluted by a gradient replacing buffer A with buffer B (1% increase/min), and elution peaks were collected. Gradient elution was necessary to obtain samples free from tetramers harboring WT EcSSB subunits [23]. Protein samples were analyzed for homogeneity using sodium dodecyl sulfate–polyacrylamide gel electrophoresis (SDS–PAGE). Samples were digested using TEV protease for His-tag removal in the same buffer. The cleaved proteins were diluted until the imidazole concentration fell below 50 mM and were applied to a Ni column (HisPur, Ni-NTA Resin). The flowthrough was then added to a Q HP (GE) anion exchange column for further purification, using a solution of 20 mM Tris–HCl (pH 8) plus 150 mM NaCl as buffer A and 20 mM Tris–HCl (pH 8) plus 1 M NaCl as buffer B in an increasing gradient of 1%/min. Eluted constructs were concentrated using Amicon Ultra centrifuge filters and stored at −80°C in a buffer containing 20 mM Tris–acetate (pH 8) and 300 mM NaCl. Absorbance spectra of samples were recorded to assess nucleic acid contamination, and absorbance values at 280 nm were used to calculate concentrations of protein variants. Protein concentrations are reported as those of tetramers throughout this article. Because the amount of Cl^−^ in samples can influence the LLPS propensity of EcSSB, stock concentrations above 300 μM were targeted in case of all constructs. This way, sample dilution resulted in residual Cl^−^ concentrations that did not affect droplet formation significantly. Purity of samples was analyzed using SDS–PAGE (Fig. 1). Minor contaminants in (GGS)_4_ and GG preparations were His-tagged forms of these constructs. RecQ helicase, dC, labeled EcSSB, and CTP were prepared as described in [8].

### Fluorescence polarization assays

Fluorescence polarization (FP) measurements were used to monitor ssDNA and protein binding by EcSSB constructs, using a BioTek Synergy H4 Hybrid Reader (excitation/emission at 485/528 nm). Assays were carried out in three independent experiments at 25°C in a buffer containing 20 mM Tris–acetate (pH 8), 50 mM K-glutamate, and 10 mM Mg-acetate. For ssDNA binding, fluorescein-labeled ssDNA oligonucleotides (36 and 79 nt, as in [24]) (Sigma–Aldrich) were applied at 10 nM and titrated with EcSSB constructs. To monitor RecQ helicase binding by EcSSB constructs, competitive FP measurements were carried out. Based on the capacity of the isolated CTP to bind RecQ [8, 15], the RecQ–fluorescein-labeled CTP (flCTP) (Sigma–Aldrich) binding affinity was determined first using direct titration measurements (25 nM flCTP titrated with increasing RecQ concentrations (0–10 μM) (Supplementary Fig. S1). RecQ–flCTP binding was also analyzed in a buffer comprising 20 mM Tris (pH 8.0), 150 mM NaCl, 10% glycerol, 1 mM ethylenediaminetetraacetic acid, and 1 mM β-mercaptoethanol, as used in [25]. During competitive measurements, a RecQ–flCTP mixture (0.65 μM RecQ, 25 nM flCTP) was titrated with EcSSB variants. Intermolecular OB domain binding abilities of EcSSB constructs were analyzed in competitive titration measurements similar to the RecQ-based assays but using dC instead of RecQ at 2.5 μM tetramer concentration (flCTP was used at 25 nM as previously). Assays were performed following the direct titration of 25 nM flCTP with increasing dC concentrations (0–20 μM) (Supplementary Fig. S1). Data were evaluated using Origin 8 (Microcal Co.), using a quadratic binding equation for direct titrations [8], and a previously established analytical framework for competitive titrations [26].

### Turbidity measurements

Condensate droplets scatter light, allowing their quantification by measuring OD_600_ of samples [8]. Dilution series of EcSSB variant samples were generated starting from a tetramer concentration of 20 μM, using the same buffer as in the case of FP assays. Turbidity measurements were performed in a TECAN Infinite F Nano+ plate reader, measuring absorbance at 600 nm (band width 10 nm) at 25°C with a 25 flash/read setting, in the absence and presence of 3% (m/v) polyethylene glycol (PEG 20K) immediately after sample preparation. Three independent measurements were carried out for each construct.

### Fluorescence microscopy

Epifluorescence microscopic images of condensate droplets were recorded using a Nikon Eclipse Ti-E TIRF microscope in epifluorescence mode. Samples were excited using a Cyan 488-nm laser (Coherent). EcSSB variants were mixed with 0.3 μM fluorescently labeled WT EcSSB before initiating condensation. Samples were measured using μ-Slide Angiogenesis (Ibidi) microscope slides at 25°C, in the same buffer conditions as in turbidity measurements. Inclusion of labeled WT EcSSB in the droplets allowed for fluorescent detection of condensates. Images were recorded 1 min after mixing unless otherwise indicated.

### Co-condensation assays

Dilutions (10 μM tetramer concentration) of each EcSSB variant, WT protein, and dC were prepared in a buffer containing 20 mM Tris–acetate (pH 8), 50 mM K-glutamate, and 10 mM Mg-acetate. Under these conditions, only WT EcSSB showed condensation. Each variant was then mixed with either WT EcSSB or dC in a 1:1 volume ratio, and the turbidity of the mixtures was monitored as described earlier for turbidity measurements. The origin of the observed turbidity changes was verified using epifluorescence microscopy.

### Condensation inhibition measurements

WT EcSSB, d1G, d2G, W136D, and dC solutions containing 20 μM tetramers, 20 mM Tris–acetate (pH 8), 50 mM K-glutamate, 10 mM Mg-acetate, and 3% (m/v) polyethylene glycol (PEG 20K) were titrated at 25°C with substances affecting EcSSB LLPS (dT_79_, NaCl, K-Glu, l-Arg), and the turbidity of samples was measured as described for turbidity assays. For WT EcSSB, a buffer lacking PEG 20K was also used. Dilution series were started from 40 μM concentration for dT_79_ and 400 mM for NaCl, K-Glu, and l-Arg. Three independent measurements were carried out for each construct.

### Temperature profiles of condensate formation

Solutions identical to those described for condensation inhibition assays were mixed at 4°C, pipetted into cuvettes with 1 cm path length, and inserted into a Cary E4 spectrophotometer with an attached water cooler thermostat. The machine was pre-cooled to 4°C. Cuvettes were sealed to prevent evaporation. Samples were heated to 60°C at a rate of 0.2°C/min, with concomitant measurement of OD_600_ in 1°C intervals. Thermal Software (version 3.0) was used for the measurements. EcSSB variants were applied at 20 μM, as some of the constructs were able to form condensates only at high protein concentration. This condition enabled direct comparison between constructs. Three independent measurements were carried out for each construct.

### Determination of component enrichment in condensates

WT, d1G, d2G, W136D, and EHE condensates were generated at 25°C in a buffer containing 20 mM Tris–acetate (pH 8), 50 mM K-glutamate, 10 mM Mg-acetate, and 10% (m/v) polyethylene glycol (PEG 20K). Elevated PEG concentration was applied as EHE forms condensates only under these conditions. Fluorescent molecules were added at 100 nM concentration following condensate formation. Samples were imaged using a Nikon Eclipse Ti-E microscope 1 and 15 min after mixing. Enrichment ratios were determined by dividing the average fluorescent intensities of 15 droplets with the average fluorescent intensities of the background next to the condensates.

### Computational predictions

AlphaFold 3 (AF3) predicted tetrameric structures were obtained using the AF3 server by default parameters and by running one EcSSB variant sequence in four copies at a time. Besides using the constructs of this study, we also used the Δ120–166, Δ130–166, and Δ151–166 variants studied by Kozlov *et al.* [9]. AF3 provided five models for each tetramer prediction by default. The confidence/quality of the models is measured by predicted template modeling (pTM) scores, where values above 0.5 are considered good. The pTM scores of all models were uniformly above 0.6 for the WT and all variants, except for the chimeric variants EPE and EHE, whose models received pTM scores between 0.54–0.56 and 0.47–0.49, respectively. Since the differences between the pTMs received for models of the same variant were subtle in each case, we did not exclude any of the models from further analysis.

LLPS predictions were carried out for the same set of variants by using seven web servers by default parameters:

1. PSPHunter [27] at http://psphunter.stemcellding.org/ using the “Predict Phase-Separating Proteins” option,
2. DeePhase [28] at https://deephase.ch.cam.ac.uk,
3. PSPredictor [29] at http://www.pkumdl.cn:8000/PSPredictor,
4. FuzDrop [30] at https://fuzdrop.bio.unipd.it/predictor,
5. PSPer [31] at https://www.bio2byte.be/b2btools/psp,
6. PScore [32] at https://pound.med.utoronto.ca/∼JFKlab/Software/psp.htm, and
7. CatGranule [33] among the tools provided at http://www.tartaglialab.com.

## Results

### EcSSB construct design for condensation engineering

To engineer EcSSB IDL-mediated condensation properties, we targeted IDL aa residues hypothesized to participate in types of interactions that have been implicated to contribute to LLPS. These include charge–charge interactions [34, 35], π-interactions [36, 37], hydrogen bonding [38, 39], and, to a lesser extent, hydrophobic contacts [40]. π-interactions are typically planar interactions between sp2-hybridized atoms (π–π interactions) of aromatic residues (Y/F/W/H), the carboxyl/carboxamide groups of N/D/Q/E, the guanidine group of R, and/or the exposed backbone peptide bond of G and other amino acids with small side chains [32]. Cation–π interactions can form between positively charged (K/R, with the latter being more favorable) and aromatic (W/Y/F) residues [21, 41, 42]. In the EcSSB IDL sequence (56 residues, aa 114–170, excluding the CTP), Q (11 instances) and G (17 instances) dominate and, together with A and S, constitute 67% of total residues in this region (with prolines representing an additional 16%). Moreover, two aromatic and two positively charged residues (R116, W136, F148, and R155) are embedded between them. Besides composition, the patterning of residues is an important determinant of LLPS propensity. Many known LLPS driving regions harbor distributed aromatic residues [37, 43], as is the case for EcSSB IDL. These findings suggest the possibility that the contributions of EcSSB IDL to LLPS are mostly mediated by π-interactions.

Based on the above considerations, we designed and produced an array of EcSSB constructs (Fig. 1). WT EcSSB and a previously produced construct lacking the CTP (dC, aa 1–170) were also used as controls. Major changes were applied in GG, (GGS)_4_, EPE, and EHE, in which the entire EcSSB IDL (except the CTP) was eliminated (GG) and changed either to a G/S-containing linker [(GGS)_4_] or to the IDL sequences of PfSSB (EPE) or hSSB1 (EHE). GG and (GGS)_4_ were designed to determine the effects of linker length between the OB domain and the CTP, while EPE and EHE were produced to elucidate the importance of the aa composition of the IDL in LLPS propensity. EPE was previously shown to be unable to phase separate, representing a robust negative control [9]. PfSSB IDL harbors a few small-side-chain amino acids and a single proline (A/S/G/P/total: 0/5/2/1/88 residues), besides the abundance of charged residues (D/E/R/K/total: 10/8/3/5/88), with the predominance of negative charges implicated to hinder LLPS via ionic repulsions. The IDL sequence of hSSB1 is more similar to EcSSB, comprising mostly small-side-chain amino acids and prolines (A/S/G/P/total: 8/10/16/23/105), with sparsely scattered charged and aromatic residues. In hSSB1, carboxamide groups are mostly in N residues (N/Q/total: 10/6/105), unlike those in EcSSB.

In addition to the earlier discussed major alterations to the IDL, specific IDL subregions were also targeted to produce variants with more subtle modifications. The importance of the IDL’s G residues was previously suggested. The IDL harbors two GGXXGGG motifs in its N-terminal region, constituting a large fraction of total IDL glycines. IDL truncated mutants harboring only the first or both motifs along with the CTP indicated that the first motif is sufficient for condensation and the second motif further enhances the process [9]. However, the specific function of the glycine-rich motifs and their interplay with other IDL regions has not been explored in detail. To test this, here we used the d1G (aa 114–120 deleted) and d2G (aa 114–130 deleted) constructs to determine the impact of these sections.

To dissect the contributions of cation–π interactions to LLPS, R116 and W136 were altered. R116 and W136 were chosen as these residues are flanked by glycines; thus, they appear more favorable for cation–π interactions (not to mention that W is more favorable compared to F in case of these connections) than F148 and R155. Earlier results by Kozlov *et al.* [9] suggested that the simple deletion of these residues might not be sufficient to eliminate condensation propensity, since deletion constructs lacking W136 (as well as F148 and R155) were able to undergo condensation. Therefore, we introduced negatively charged residues in these positions (W136D and R116D–W136D constructs), which may introduce ionic repulsions between IDLs besides eliminating cation–π interactions.

We expressed and purified all mentioned EcSSB variants to determine their ssDNA and protein partner binding abilities alongside their LLPS propensity.

### IDL-engineered EcSSB constructs retain the capacity for binding ssDNA and RecQ helicase

**Figure 2.**
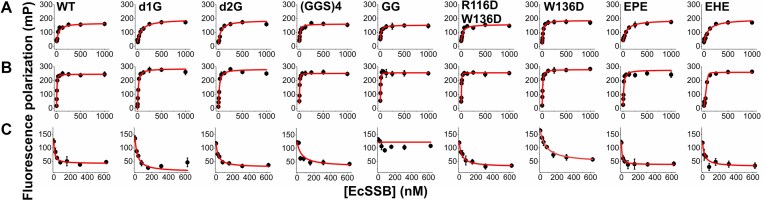
ssDNA and RecQ helicase binding by IDL-engineered EcSSB constructs. FP titrations of 10 nM labeled ssDNA oligonucleotides (**A**, ssDNA_36_; **B**, dT_79_) with EcSSB constructs (tetramer concentrations stated throughout the article) showed retained ssDNA binding capability for all constructs. Lines show best fits based on a quadratic binding equation (see the “Materials and methods” section and Supplementary Table S1). (**C**) Twenty-five nanomolar labeled SSB C-terminal peptide (flCTP) was complexed with RecQ helicase (650 nM) and titrated with EcSSB constructs in competitive FP assays. Lines show best fits based on a competitive binding scheme ([26], see also the “Materials and methods” section and Supplementary Table S1). Data points show the mean ± standard deviation (SD) of three independent experiments in each case.

As part of the functional characterization of the engineered EcSSB variants, we carried out ssDNA binding and PPI measurements. As EcSSB has two major ssDNA binding modes associating with 35 and 65 nt on ssDNA (SSB_35_ and SSB_65_, respectively) [24], we applied two fluorescently labeled ssDNA species that were used also in our earlier study (flssDNA_36_ and fldT_79_) [8] to elicit these two binding modes. Note that these binding modes are nevertheless dictated not only by DNA size but also by other factors (e.g. ion types and concentrations). We found that the interaction of EcSSB with these ssDNA ligands was unaffected by IDL modifications (Fig. 2A and B, Supplementary Table S1), supporting that the OB domain is specifically and exclusively responsible for these interactions [14].

Besides binding to ssDNA, SSBs are PPI hubs promoting the assembly of genome maintenance complexes on genomic target sites [14, 16, 44]. Therefore, we assessed the binding of EcSSB variants to RecQ helicase, a key SSB partner [25, 45]. All EcSSB constructs, except GG, showed WT-like RecQ binding affinities (Fig. 2C and Supplementary Table S1). It is known from previous studies that dC is unable to bind RecQ since it is missing crucial residues for binding [15]. These results show the importance of the steric availability of the CTP (which is hindered in the GG construct) for the binding of RecQ helicase (and supposedly for the binding of other protein partners). Taken together, our results indicate that the IDL can be readily engineered without affecting interactions to partner proteins and to ssDNA.

### The condensation propensity of EcSSB can be influenced in a broad range via IDL engineering

**Figure 3.**
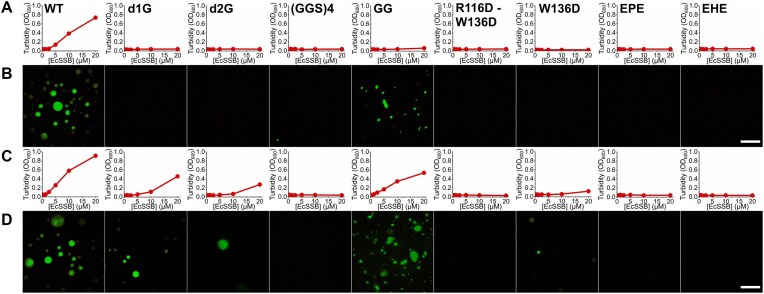
IDL engineering markedly alters the condensation propensity of EcSSB constructs. Turbidity (OD_600_) measurements were carried out in the (**A**) absence or (**C**) presence of molecular crowder [3% (m/v) PEG 20K]. Each data point represents the mean ± SD of three independent experiments. Error bars are within symbols. To determine whether the observed turbidity increase is caused by LLPS-based condensation or amorphous aggregation, epifluorescence microscopic images were recorded in the (**B**) absence or (**D**) presence of 3% PEG 20K. Twenty micromolar of each EcSSB construct was mixed with 0.3 μM fluorescently labeled WT EcSSB before imaging. Spherical droplets indicate LLPS (cf. those in WT samples), whereas amorphous aggregates were seen in GG samples. Scale bars, 20 μm.

Despite the retained ssDNA and partner protein binding functionalities (Fig. 2), none of the earlier described EcSSB variants were able to form condensates in the absence of molecular crowder, in contrast to WT EcSSB (Fig. 3). Even in the presence of 3% (m/v) PEG 20K (to better mimic the cellular environment [46–48]), only d1G, d2G, and W136D showed moderate propensity for condensation (Fig. 3). In line with what has been shown previously using bovine serum albumin as crowder [8], dC was also able to form condensates in the presence of 3% PEG 20K (Supplementary Fig. S2). For all condensation-competent constructs, turbidity values increased with increasing PEG 20K concentration (Supplementary Fig. S2). The size of EcSSB droplets also increased with PEG 20K concentration, presumably due to the surface activity of PEG (Supplementary Fig. S3) [49]. EPE and R116D–W136D were unable to form condensates throughout the examined PEG 20K concentration range (Supplementary Fig. S2). Interestingly, EHE started to show propensity for condensation at 7.5% PEG 20K and at high protein concentration (20 μM; EcSSB concentrations are specified as those of tetramers throughout this article). The IDL of hSSB1, contained in EHE, shows a similar aa composition to that of EcSSB (Fig. 1). However, these results show that its sequence confers a reduced capability for driving condensation in the context of EcSSB. We also found that the GG construct, which lacks the IDL, forms amorphous aggregates at high protein concentrations or in the presence of a crowding agent (Fig. 3).

Taken together, the above results shed new light on the contribution of the IDL region to the condensation propensity of EcSSB. The data show that, while OB domain–CTP interactions are important in enhancing condensate formation, the condensation propensity can be dominantly influenced and precisely engineered through IDL modifications.

### IDL compatibility governs co-condensation of EcSSB variants

**Figure 4.**
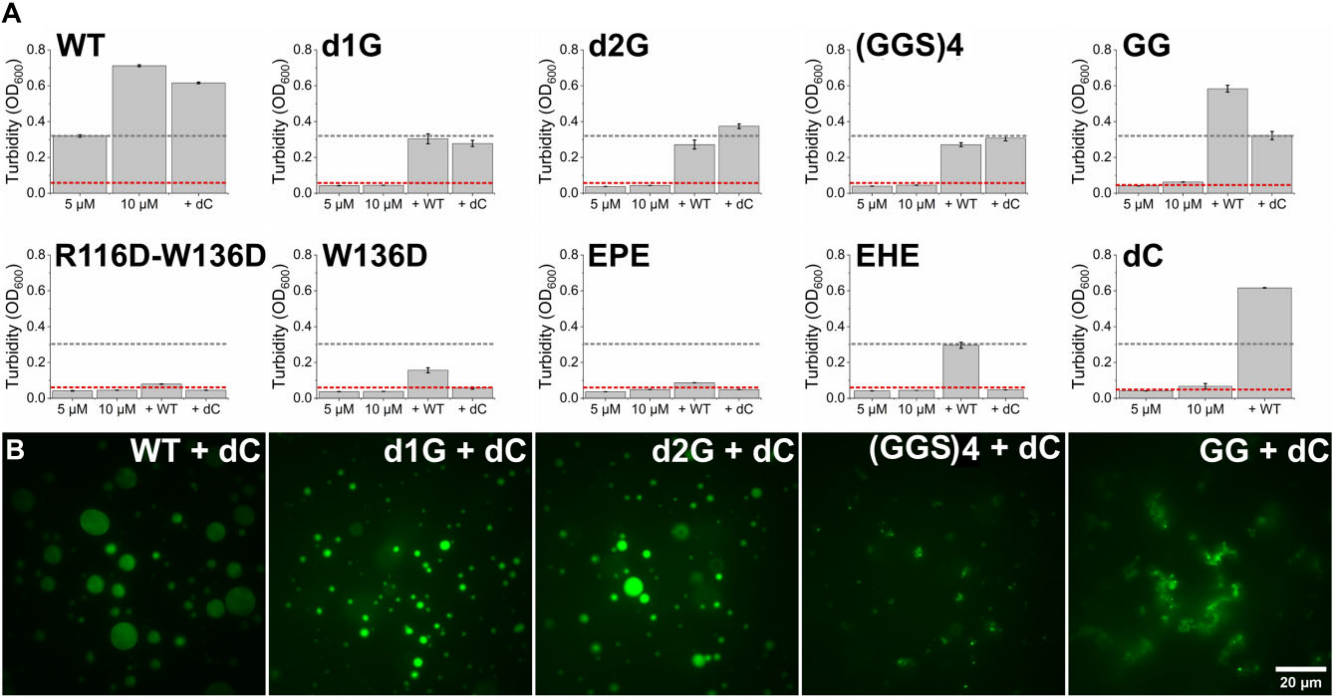
Co-condensation ability of EcSSB constructs with the WT protein and the dC variant in the absence of molecular crowder. (**A**) Samples contained 5 or 10 μM of each EcSSB construct as indicated for the first two columns of each panel. “+ WT” and “+ dC” columns show turbidity values of 10 μM target construct mixed with 10 μM WT or dC, respectively, at 1:1 volume ratio (thus resulting in 10 μM total protein concentration). Gray and red dashed lines show the turbidity of 5 μM WT and dC constructs, respectively, providing references for co-condensation effects. “+ WT” values above the gray line, or “+ dC” values above the red line, are indicative of effective co-condensation. Each column represents the mean ± SD of three independent experiments. (**B**) Co-condensates imaged upon mixing 10 μM EcSSB variant (as indicated in each panel) with 10 μM dC at 1:1 volume ratio (thus resulting in 10 μM total protein concentration). Condensates were visualized using 0.3 μM fluorescently labeled WT EcSSB.

Next, we sought to determine whether the presence of WT EcSSB can enhance condensate formation by the IDL-engineered EcSSB constructs, and/or the IDL-engineered (CTP-containing) constructs can enhance condensate formation by dC. To this end, we performed turbidity assays using mixtures of EcSSB constructs with WT or dC (Fig. 4). We found that WT EcSSB can readily recruit dC molecules into the condensates in line with our previous results [8], and the two proteins act in an additive manner. This finding suggests that the identical IDLs of the two constructs can naturally assemble into condensates. However, the WT protein did not show additive co-condensation with any other constructs investigated and the increased turbidity in case of GG was caused by increasing level of aggregates. Remarkably, R116D–W136D, W136D, and EPE even had a strong inhibitory effect on WT EcSSB condensation. Moreover, similar to WT, d1G and d2G were able to restore the condensation propensity of dC in the absence of crowder, while (GGS)_4_ and GG formed amorphous aggregates with dC. Note that GG was found to also form aggregates alone; however, (GGS)_4_ remains in solution when dC is not present (Fig. 3). At the same time, the condensation propensity of R116D–W136D, W136D, EPE, and EHE could not be rescued by the presence of dC. Together, these results show that the length and composition of the IDL are critical in determining the compatibility of intertetramer interactions leading to condensate formation and greatly affect the availability of the CTP for intertetramer OB–CTP interactions.

### CTP exposure is greatly influenced by IDL structure, but does not determine condensation propensity

To study the molecular background of the above co-condensation features, we assessed the interaction of the EcSSB OB domain with the CTP. First, we measured the direct binding of a labeled isolated CTP peptide (flCTP) to EcSSB constructs (direct FP measurement). We found that flCTP can bind to dC with a *K*_d_ of 2.1 ± 1.0 μM (Supplementary Fig. S1), while the endogenous CTP contained in other constructs efficiently competed with the free peptide, leading to very weak flCTP binding (e.g. *K*_d_ > 50 μM). These data explain enhanced co-condensation of dC with WT, d1G, and d2G (Fig. 4), all of which can provide CTP segments to interact with the OB domain of dC. However, the above CTP binding data do not explain the inability of all other assessed constructs (EPE, EHE, W136D, and R116D–W136D) to co-condense with dC (Fig. 4), pointing to the dominant role of IDL compatibility.

**Figure 5.**
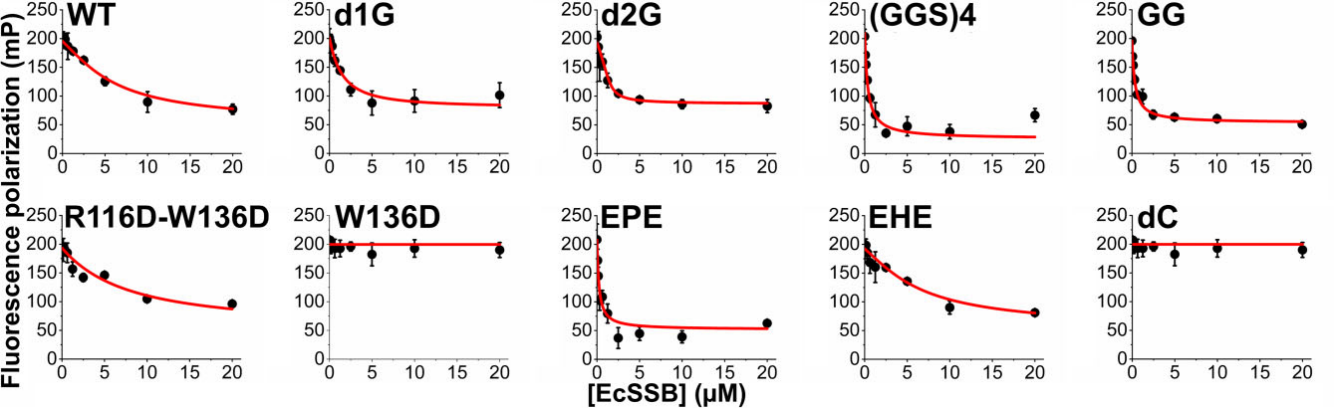
Intermolecular OB domain binding by CTP segments of EcSSB constructs, monitored in competitive titrations. Fluorescein-labeled SSB C-terminal peptide (flCTP, 25 nM) was complexed with dC (2.5 μM) (*K*_d_ = 2.1 ± 1.0 μM, cf. Supplementary Fig. S1), and this complex was titrated with the indicated EcSSB constructs in competitive FP assays. Decrease in FP values reveals the ability of the CTP segment of each EcSSB construct to compete with flCTP for binding to the OB fold of dC tetramers. Data points represent the mean ± SD of three independent experiments. Lines show best fits based on a competitive binding scheme ([26], see also the “Materials and methods” section and Supplementary Table S1).

We assessed the ability of each EcSSB construct’s CTP segment to compete for binding to the OB domain of dC in competitive FP assays in which a pre-formed complex of dC and the isolated flCTP peptide was titrated with increasing concentrations of each EcSSB construct (Fig. 5 and Supplementary Table S1). In general, we found an increasing tendency in CTP exposure (competition ability) with decreasing IDL length: R116D–W116D and EHE showed WT-like competition ability, whereas this feature was markedly enhanced in d1G and d2G, and even more strongly enhanced in (GGS)_4_ and GG. EPE did not follow this tendency, by having WT-like IDL length but greatly enhanced competition ability. This is presumably due to the formation of strong interactions between the negatively charged sequence of the PfSSB IDL, contained in EPE, and the dC OB domain. W136D represented another outlier, which completely lacked competition ability despite its IDL length being identical to WT EcSSB. This finding suggests that W136D adopts divergent IDL conformation(s) that prevent its CTP from being able to compete for binding to dC’s OB domain. Together with the greatly diminished condensation propensities of the IDL-engineered constructs (Fig. 3), these data show that CTP exposure alone does not determine EcSSB condensation propensity, which is greatly influenced by IDL conformation and IDL–IDL interactions.

### IDL-engineered EcSSB variants retain ssDNA regulation but show impaired stability of condensates

**Figure 6.**
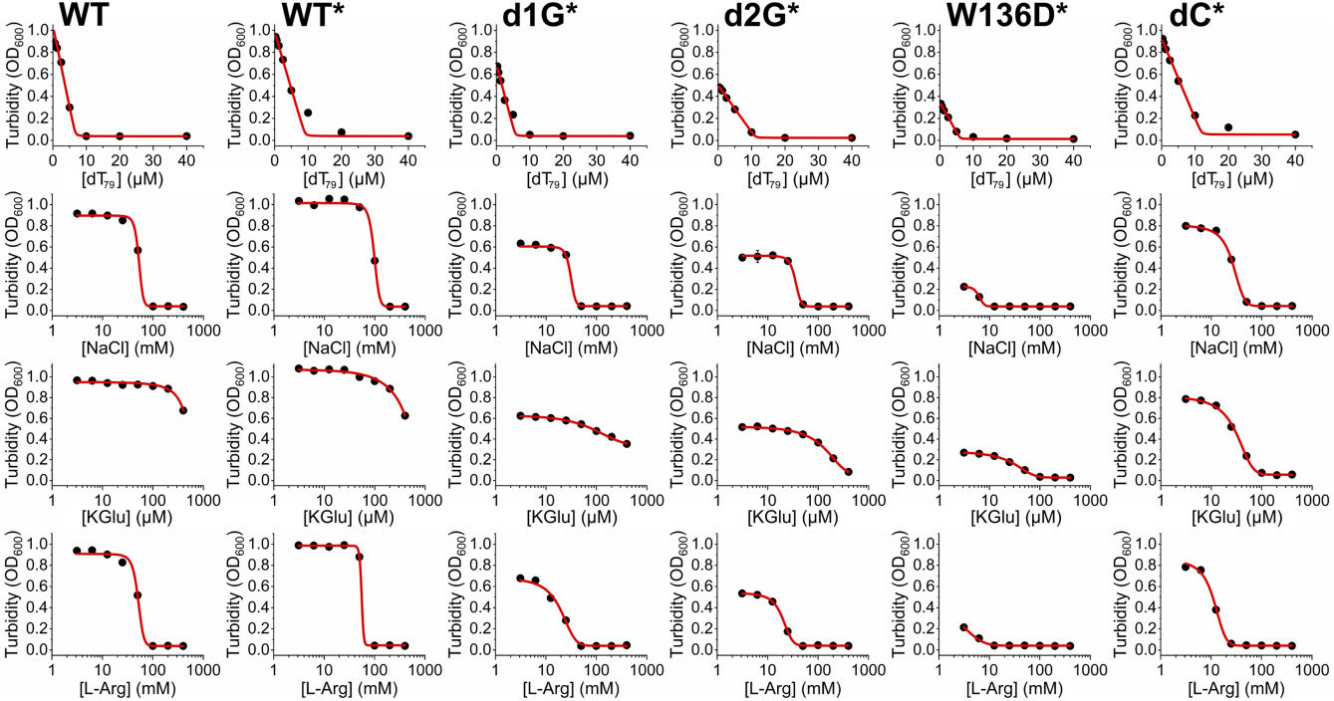
Inhibition of condensation of EcSSB variants by ssDNA and ionic conditions. Condensates of indicated EcSSB constructs (20 μM) were titrated with ssDNA (dT_79_), NaCl, K-Glu, and l-Arg to determine their effects on EcSSB condensation. Asterisks (*) show the presence of 3% (m/v) PEG 20K molecular crowder in the experiments. Note that all variants used here, except for WT, required the presence of crowder for condensation (cf. Fig. 3). Data points represent the mean ± SD of three independent experiments. Error bars are within symbols. Lines show best fits based on the quadratic binding equation [8] in case of dT_79_ and Hill equation in case of others (see also Supplementary Table S2).

Stoichiometric inhibition by ssDNA was reported as a defining feature of WT EcSSB condensation, suggesting its *in vivo* role in rapid intracellular EcSSB redistribution upon the appearance of ssDNA segments during genome damage [8]. Here, we found this feature to be unaffected by IDL engineering, in accordance with the WT-like ssDNA binding capability of the engineered EcSSB variants (Fig. 6 and Supplementary Table S2; cf. Fig. 2 and Supplementary Table S1).

We monitored the stability of EcSSB condensates via dose-dependent inhibition of condensation by NaCl, K-Glu, and l-Arg (pH 8) (Fig. 6 and Supplementary Table S2). EcSSB condensation was previously shown to be potently inhibited by supraphysiological concentrations of chloride ions [8]; here, l-Arg proved to be a similarly competent condensation inhibitor, presumably blocking cation–π interactions. We found increased sensitivity for all assessed engineered EcSSB constructs to chloride and l-Arg compared to WT EcSSB (Fig. 6 and Supplementary Table S2). Glutamate was shown to promote WT EcSSB condensation at physiological and inhibit condensation at supraphysiological high concentrations [8, 9]. Here, we found that d1G retained the WT-like sensitivity for Glu, but all other assessed constructs showed enhanced Glu sensitivity of condensation (Fig. 6 and Supplementary Table S2).

**Figure 7.**
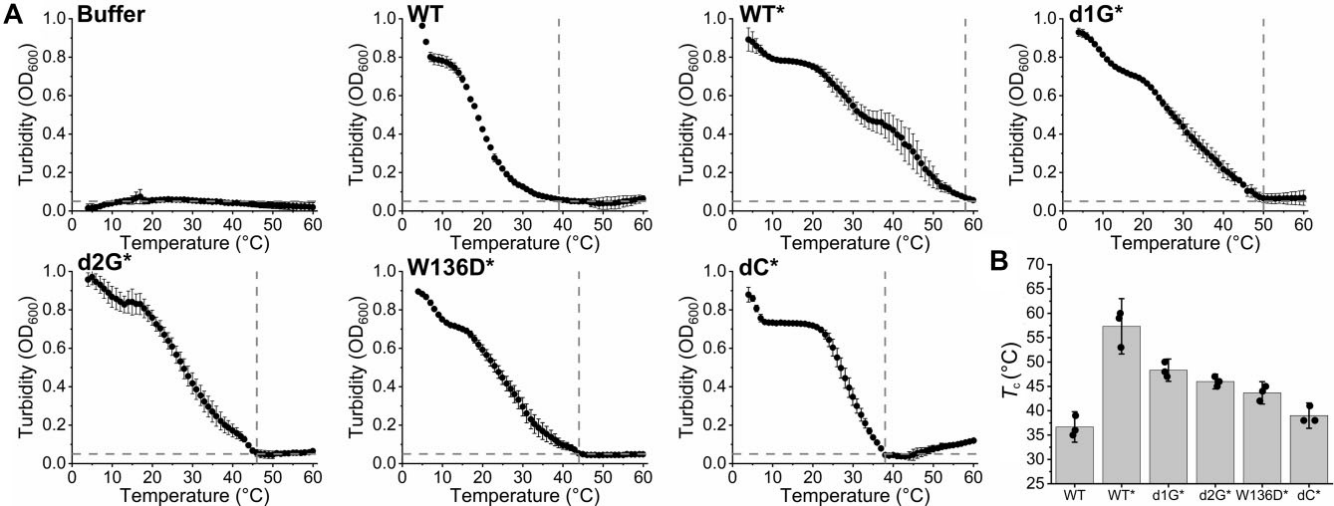
Temperature profiles of condensate formation by EcSSB constructs reflect modulation of condensation propensity. (**A**) Temperature profiles of turbidity (OD_600_) values of samples of indicated EcSSB constructs (20 μM) are shown. Samples were heated from 4°C to 60°C at a rate of 0.2°C/min. Data points represent the mean ± SD of three independent experiments. The horizontal dashed line shows the reference (buffer) condensation-free absorbance value, while vertical lines mark temperatures at which each EcSSB variant reached the condensation-free value (*T*_c_). Profiles marked with * were recorded in the presence of 3% (m/v) PEG 20K molecular crowder. (**B**) *T*_c_ values determined from experiments in panel (A). Columns show the mean ± SD, with individual experimental values shown as dots.

Temperature dependence of condensation, monitored through turbidity, was previously established as another useful quantitative measure of condensate stability [9]. Upon controlled slow heating of EcSSB samples in the presence of molecular crowder, we found that the condensation-inhibiting temperature (*T*_c_, at which the samples reached the condensate-free turbidity value) was gradually reduced in the order of WT > d1G > d2G > W136D > dC (Fig. 7). Importantly, we found that the presence of crowder greatly increased the temperature resistance of WT EcSSB condensates, implying an important condensate-stabilizing effect of the cellular environment (Fig. 7). The multiphasic nature of curves, especially in crowder-containing samples, probably reflects contributions from multiple types of interactions between EcSSB tetramers and/or solution components.

### Enrichment of EcSSB binding partners in condensates can be retained despite impaired condensate formation

**Figure 8.**
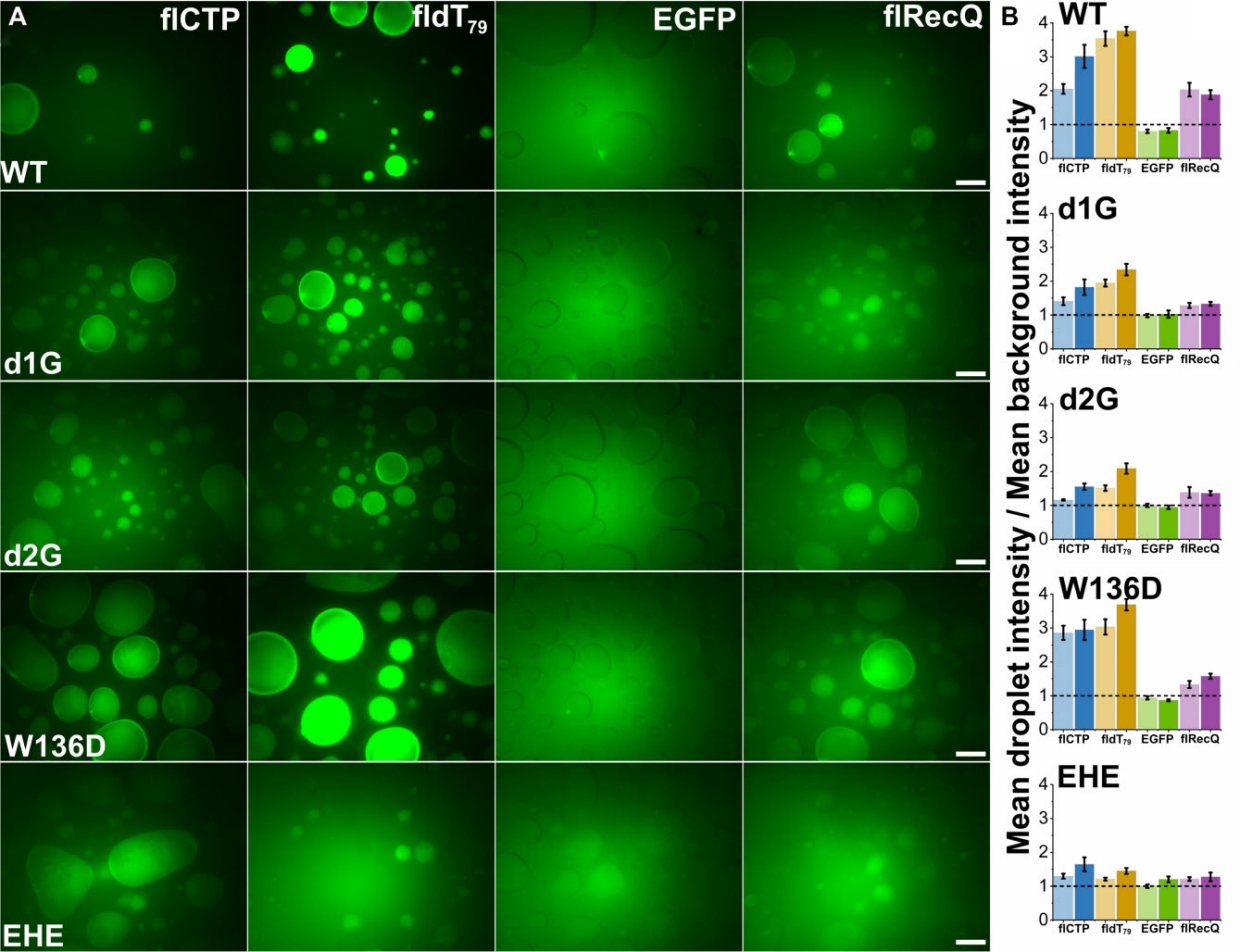
EcSSB constructs show diverse profiles for enrichment of binding partners inside condensates. (**A**) Epifluorescence images indicating the extent of enrichment of different fluorescently labeled partner/control molecules in condensates of indicated EcSSB constructs (20 μM) 15 min after mixing. In these experiments, 10% (m/v) PEG 20K was applied to minimize the amount of dissolved (non-condensed) EcSSB molecules, thereby reducing background fluorescence. Scale bars, 20 μm. (**B**) Enrichment (mean droplet intensity/mean background intensity) values of fluorescent molecules in EcSSB condensates (flCTP, blue; fldT79, sand; eGFP as negative control, green; flRecQ, purple). Light and dark colors indicate results obtained after incubating EcSSB condensates with the fluorescent partner/control molecules for 1 and 15 min, respectively. Values >1 (dashed lines) indicate enrichment of the labeled molecules in EcSSB condensates. Mean ± standard error of the mean values for *n* = 15 are shown.

The enrichment of binding partners inside EcSSB condensates is another feature with proposed physiological importance in organizing genome repair complexes [8]. Here, we assessed this feature by adding 100 nM fluorescently labeled partner/control molecules [ssDNA, isolated EcSSB CTP, RecQ helicase, and enhanced green fluorescent protein (eGFP) as a negative control] to condensates formed by EcSSB constructs. Droplets were imaged 1 and 15 min after mixing. We monitored the signal ratio of fluorescent partners within versus outside EcSSB condensates to determine enrichment (Fig. 8). We found that the W136D construct generally retained the partner enrichment features (Fig. 8), despite its impaired condensation properties (Figs 3, 4, and 7). The other assessed engineered constructs showed weakened enrichment of partners, especially for RecQ helicase (Fig. 8), despite the fact that they could bind RecQ with WT-like affinities (Fig. 2C). Data show that partner enrichment features can be engineered separately from condensation propensity.

### AF3 predicted tetrameric structures of EcSSB reveal versatile modes of CTP binding to the OB domain and provide structural insights into ssDNA regulation of EcSSB condensation

**Figure 9.**
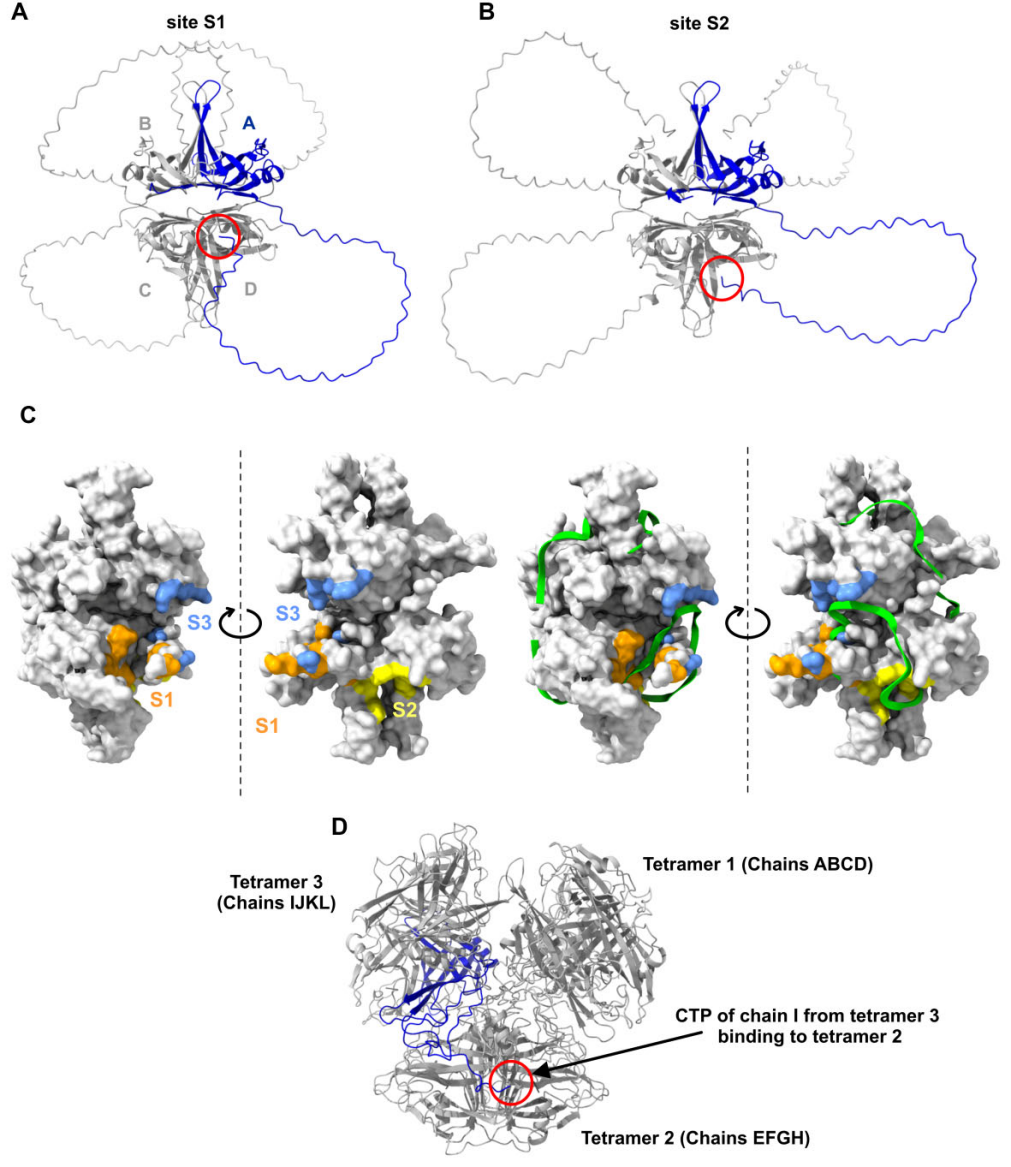
Intra- and intertetramer OB–CTP interactions in EcSSB identified by AF3. (**A**) Tetrameric model structure of WT #0 (cf. Supplementary Table S3) in cartoon representation, showing the CTP (encircled in red) of chain A (blue) binding to site S1 (highlighted in orange in panel (C) and in Supplementary Table S3) on the tetramer surface of the opposite dimer (chains CD) in “*trans*” configuration (cf. Supplementary Table S3). (**B**) Tetrameric model structure of WT #2, showing the CTP binding to site S2 (highlighted in yellow in panel (C) and in Supplementary Table S3) in “*trans*” configuration. (**C**) Key residues within sites S1, S2, and S3 (orange, yellow, and blue, respectively; cf. Supplementary Table S3) mapped onto the EcSSB OB tetramer surface (PDB: 1EYG) with the bound ssDNA segment hidden (left) or shown (green, right). It is apparent from the models that ssDNA occupies all predicted CTP binding sites. (**D**) Model WT #0 of the AF3 prediction encompassing 12 EcSSB WT chains depicting three tetramers, with the CTP of chain I of tetramer 3 (blue) forming an intertetramer OB–CTP interaction with tetramer 2.

We obtained AF3 predictions [50] for the tetrameric forms of EcSSB constructs used in this study and in [9] to gain insights into possible intra- and intertetramer interactions as well as conformational features that could underlie the observed varying condensation propensities. The predictions generated five models for each variant, with similar confidence values (Fig. 9A–C, Supplementary Table S3, and Supplementary Data File S1; see also the “Materials and methods” section). The CTP bound back onto the OB tetramer in four of the five models for EcSSB WT, in agreement with earlier results showing that the CTP can interact with the OB tetramer, but it is highly mobile [51, 52]. The models delineated two discernible CTP binding sites on the OB tetramer. The major site (S1, orange in Fig. 9C and Supplementary Table S3), represented in three of the five WT models, lies at the OB dimer interface (the OB tetramer is a dimer of dimers) and is primarily defined by residues F61, R85, and V106 of one monomer and residues W41 and K44 of the other monomer. Corroborating the validity of the predictions, we found that in many S1 involving models, F172 of the CTP contacts V30 and V59, the two OB residues that were identified to mediate CTP interactions by nuclear magnetic resonance (NMR) [51]. Therefore, we conclude that S1 in the AF3 models is identical to the CTP binding site detected by NMR previously with HSQC analysis (Fig. 9 and Supplementary Table S3). We also identified a second, novel CTP binding site on the OB dimer interface, defined by residues T86 and Y98 from one monomer and residues T34, W55, and R57 from the other monomer (S2, yellow in Fig. 9C and Supplementary Table S3). The identity of S2 was supported by its occupation by CTP in one of the WT models and in numerous models of the IDL-engineered variants (Fig. 9C and Supplementary Table S3). S1 is clearly occupied by ssDNA in the ssDNA-bound EcSSB crystal structure (1EYG, in agreement with the authors’ claim in [51]), as two key S1 residues, R85 and V106, form direct H-bonds with ssDNA, and most other S1 residues are categorized as interfacing (Fig. 9C). Similarly, T86 of S2 is H-bonded to ssDNA and the other key S2 residues are also interfacing (Fig. 9C). These computational predictions point to the importance of competition between ssDNA and the CTP for binding sites on the OB domain, which was proposed as a major contributor to the ssDNA regulation of EcSSB condensation [8]. Moreover, in the ssDNA-bound state of SSB, the CTP becomes more available for intermolecular interactions with its various protein partners. AF3 results obtained for engineered EcSSB variants are further discussed in the legend to Supplementary Table S3.

The flexibility of the IDL allowed for different OB–CTP binding configurations in the models, including both “*trans*” (where the CTP of chain A binds to the other dimer made up of chains C and D) and “*cis*” configurations (where the CTP binds within the dimer) (Supplementary Table S3). These findings reinforce that, given the presence of four CTPs and at least four CTP binding sites per tetramer (S1 and S2 in each OB dimer), SSB tetramers represent an optimal system for the formation of multivalent weak interactions that are the cornerstones of LLPS. In addition, weak interactions between the IDLs as well as intertetramer OB–CTP and IDL–IDL interactions are likely to further increase the LLPS propensity of EcSSB.

In addition to the earlier discussed single-tetramer structures, we also generated predicted structures using 8, 12, or 16 copies of EcSSB WT to gain insights into possible intertetramer interactions (Supplementary Data File S2). These multitetramer structures received low confidence scores from AF3 (pTMs were between 0.25 and 0.32), and some of the resulting models were clearly unrealistic (e.g. when 16 chains were used, the tetramers fell apart). Nonetheless, the highest-scoring model of the 12-chain prediction depicted three tetramers that formed identifiable intertetramer OB–CTP interactions (Fig. 9D).

### Condensation engineering data support the development of LLPS prediction algorithms

The availability of experimental data on the condensation properties of a comprehensive set of engineered EcSSB variants (this study and [9]) also enabled the comparative assessment of the accuracy of available LLPS prediction methods, including PSPHunter [27], DeePhase [28], PSPredictor [29], FuzDrop [30], PSPer [31], PScore [32], and CatGranule [33] (Supplementary Table S4). As expected, second-generation predictors (PSPHunter, DeePhase, PSPredictor, and FuzDrop) that were developed after the publication of dedicated LLPS databases showed better performance compared to first-generation methods (PSPer, PScore, and CatGranule) that had limited training data and are often specialized for a particular LLPS mechanism (see the legend to Supplementary Table S4 for details). In summary, although some currently available LLPS prediction methods correctly assigned decreased LLPS propensity to variants with large deletions or segment changes, the extent of change is generally not predicted accurately and most predictors are insensitive to more subtle sequence changes, even if such changes markedly affect LLPS propensity. Moreover, all methods seem to suffer from a disorder bias, i.e. they assign an LLPS-promoting effect to all IDRs and thus give erroneous predictions for IDR variants that negatively affect LLPS by any mechanism. These shortcomings, as well as the strong linkage of protein LLPS to human diseases, highlight the pressing need for more accurate LLPS prediction methods that can reliably estimate the effects of even subtle sequence changes (such as disease-associated mutations) and identify key modifications required to modulate LLPS processes in a desired way. Experimental LLPS engineering datasets, such as those presented in the current study, will be indispensable for the training of such improved methods, especially if collected in and converted to a unified format that is ideal for method training.

## Discussion

Despite its emerging central roles in bacterial physiology, protein condensation in bacteria is still poorly explored [6, 7]. The condensation propensity of EcSSB was recently demonstrated and characterized, but its function in living cells is still unclear [8, 9]. EcSSB is vital for *E. coli* cells due to its multifaceted nucleic acid stabilizing and protein interaction organizing functions, associated chiefly with its OB domain [11, 53] and CTP segment [12, 54, 55], respectively. The SSB IDR appears dispensable for the growth of isogenic bacterial populations under stress-free laboratory conditions in nutrient-rich media. SSB variants harboring even large deletions within the IDR can restore viability in the lethal SSB knockout background [56, 57]. Importantly, however, evidence has recently been accumulating for an emerging substantial role of the SSB IDR—and, possibly, SSB’s IDR-mediated condensation propensity—in supporting long-term (competitive) fitness through maintaining genome stability and possibly additional cellular stress response mechanisms (see below). Perturbations in IDR structure and, in turn, in associated SSB functions, confer measurable disadvantages to cells in stressed and/or heterogeneous bacterial populations that more closely mimic real-life circumstances.

- In the work of Bonde *et al.* [12], *E. coli* cell lines harboring IDR-shortened SSB variants showed normal growth under unstressed conditions. However, reductions in UV survival were detected that were proportional to the extent of IDR deletion. Importantly, the SSB variant with the shortest IDR assessed (d120–166, similar to the GG construct used by us in the current work) conferred a marked disadvantage in competitive growth over a 3-day period relative to the WT strain. This genotype also showed a small but detectable level of basal (constitutive) SOS DNA damage response induction.
- Furthermore, in the follow-up study of Sandler *et al.* [58], all of the above-mentioned IDR-modified SSB variants produced synthetic lethality with *priA* knockout. Intriguingly, the d120–166 construct (alongside a variant harboring a large insertion within the IDR) additionally conferred synthetic lethality with priB knockout or an amino acid-substituted PriA (C479Y) variant. Notably, cells harboring SSB d120–166 showed a small-colony phenotype in different *priA* point mutant or RecBCD-deficient or RuvABC-deficient backgrounds.
- Antony *et al.* [45] found that *E. coli* cells expressing an engineered SSB variant in which an SSB tetramer harbors only two native IDR sequences (including the conserved CTP) show accelerated accumulation of mutations.
- Although not directly targeting the IDR, the results of Feliciello *et al.* [59] further underscore the role of SSB-mediated molecular functions in long-term genome stability. The *ssb-1* (H55Y amino acid substituted) mutant showed a marked (>100-fold) increase in the frequency of illegitimate recombination at nonpermissive temperature. This effect was cumulative in relation to the ablation of the genome maintenance enzyme RecQ helicase, pointing to significant nonoverlapping functions besides the known interaction between these proteins.
- A comprehensive assessment of independently evolved lineages of endosymbiont and intracellular pathogenic bacteria revealed that the SSB IDR is one of a very limited number of IDRs commonly retained by these evolutionary lineages whose proteomes are generally depleted for IDRs [60]. This result represents an additional implication for the substantial role of the SSB IDR in sustaining long-term fitness of bacteria.

The above findings imply that, besides OB- and CTP-mediated functions, the IDL emerges as a promising target for functional dissection of the cellular roles of EcSSB’s dynamic condensation, in line with the general condensation-driving propensity of intrinsically disordered protein regions. Our results demonstrate that the condensation property of EcSSB can be selectively modified or abolished by engineering the IDL, independent of other molecular functions. We find EcSSB condensation to be highly sensitive to deletions close to the OB domain (in the N-terminal part of IDL) and modifications affecting aromatic and/or positively charged residues in the IDL (Figs 1–3). Notably, Kozlov *et al.* [9] reported retained (WT-like) condensation propensities for EcSSB constructs harboring large deletions in the C-terminal part of the IDL (constructs Δ130–166 and Δ151–166) and found impaired condensation propensity only in the case of the largest deletions (Δ120–166 and Δ113–166). From these results, they suggested the importance of glycine-rich regions of the IDL in condensation; however, this feature was not assessed previously for EcSSB variants with selectively engineered GGXXGGG motifs. Here, we show that the glycine-rich regions of the IDL are indeed influential for condensation, as their selective elimination in the d1G and d2G variants resulted in impaired condensation that was not observed upon deletion of the C-terminal parts of the IDL. Notably, d1G and d2G exhibit crowder-assisted condensation, suggesting that the glycine-rich regions are important but not essential enhancers of condensation, as also found for the CTP segment here and previously [8, 9].

The Δ120–166 and Δ130–166 constructs of Kozlov *et al.* were still able to form condensates, showing that W136 alone is dispensable for condensation [9]. However, here we found that the substitution of W136 by a negatively charged residue in the W136D construct severely impairs condensation (Figs 1–3), presumably by introducing ionic repulsion between IDLs and/or major changes in the conformational distribution of the IDL, as suggested by the altered SDS–PAGE mobility of the W136D construct compared to WT EcSSB, which is even more apparent in the case of the R116D–W136D double substituted variant (Fig. 1C).

Strikingly, the introduction of two D residues in the double-substituted R116D–W136D variant completely abolished condensation even in the presence of molecular crowder (Fig. 3). These findings show that these fine-tuned modifications to the IDL result in a similar drastic condensation abolition effect as that seen for the EPE construct harboring a major IDL alteration (Fig. 3, in line with the results of [9]). Thus, R116D–W136D emerges as a suitable candidate for *in vivo* condensation engineering, unlike EPE, in which other EcSSB functions are also affected, as we showed in case of intermolecular OB domain binding experiments (Fig. 5).

In this work, we also demonstrate that condensate stability, sensitivity to ionic conditions, and binding partner enrichment can be selectively fine-tuned via IDL engineering (Figs 6–8). Of all constructs assessed, WT EcSSB condensates showed the highest stability and greatest extents of partner enrichment, reflecting precise adaptive fine tuning of the natural IDL sequence. As for the engineered variants, on one hand, W136D condensates showed the lowest stability, but their partner enrichment ability remained similar to that of WT condensates (Fig. 8). On the other hand, the d2G construct formed more stable condensates, but impaired partner enrichment capability compared to W136D. Moreover, EHE can barely form condensates only when assisted by high crowder concentrations, and its condensates also lack partner enrichment capability (Fig. 8). These data point to the utility of these constructs in future studies aimed at *in vivo* dissection of the mentioned functional properties. Our detailed quantitative assessment of the enrichment of ssDNA, RecQ helicase, and the isolated CTP peptide in EcSSB condensates (Fig. 8) shows that enrichment is controlled not only by the accessibility of the respective interaction surfaces on EcSSB constructs, but also by partner affinity and molecular size. ssDNA and RecQ helicase bind to EcSSB with similar affinities, while CTP is a much weaker binder (Fig. 2, Supplementary Fig. S1, and Supplementary Table S1). They vary also in size, CTP being the smallest (∼1 kDa), followed by dT_79_ ssDNA used in this study (28 kDa) and RecQ helicase (82 kDa). Our data revealed that partners showing the highest levels of enrichment are either highly specific binders (ssDNA) for which the binding energy drives enrichment, or small molecules (CTP) that can move more freely, causing a lower entropic cost associated with partner enrichment [61].

AF3 predictions for WT EcSSB revealed two different binding sites of the CTP on the OB tetramer (one of them matching the site previously detected by NMR) [51] as well as unbound CTP states, and imply that the two OB-bound sites can be approached in versatile binding modes and both in *cis* and *trans* configurations (Fig. 9 and Supplementary Table S3). These findings underline that EcSSB tetramers represent ideal systems for dynamically forming multivalent weak interactions, which is a cornerstone of LLPS. AF3 models of the IDL-engineered variants suggest that most of the tested IDL manipulations result in reduced IDL dynamics/mobility that typically manifest in more restricted binding site and/or configurational preferences compared to those observed for WT SSB. Such restrictions lead to reduced dynamics and multivalency in the system, which explains the lack of or reduced LLPS observed for most variants. Besides providing a rational explanation for the reduced LLPS of IDL-engineered variants, AF3 models complement IDL engineering results and underline that the sequence and structure of the N-terminal IDL segment is a major determinant in the intra- and possibly intermolecular binding preferences of the CTP and in phase separation.

All in all, our data point to a range of opportunities inherent in condensate engineering via precise modification of intrinsically disordered regions. These studies (i) enable the functional dissection of *in vivo* condensation features, (ii) aid the better understanding of the structural determinants of pathological condensation leading to human disease [62, 63], (iii) may underpin the development of therapeutic agents as well as biotechnological applications, and (iv) provide indispensable training data for future LLPS prediction methods, enabling increased accuracy and sensitivity in mutation effect prediction as well as in the computational support of LLPS engineering endeavors.

## Supplementary Material

gkaf481_Supplemental_File

## Data Availability

The data underlying this article are available in the article and in its online supplementary material. The data underlying this article will be shared on reasonable request to the corresponding author.

## References

[1] Nowicka B, Kruk J Powered by light: phototrophy and photosynthesis in prokaryotes and its evolution. Microbiol Res. 2016; 186–187:99–118. 10.1016/j.micres.2016.04.001. 27242148

[2] Shin Y, Brangwynne CP Liquid phase condensation in cell physiology and disease. Science. 2017; 357:eaaf4382 10.1126/science.aaf4382. 28935776

[3] Banani SF, Lee HO, Hyman AA et al. Biomolecular condensates: organizers of cellular biochemistry. Nat Rev Mol Cell Biol. 2017; 18:285–98. 10.1038/nrm.2017.7. 28225081 PMC7434221

[4] Hirose T, Ninomiya K, Nakagawa S et al. A guide to membraneless organelles and their various roles in gene regulation. Nat Rev Mol Cell Biol. 2023; 24:288–304. 10.1038/s41580-022-00558-8. 36424481

[5] Spannl S, Tereshchenko M, Mastromarco GJ et al. Biomolecular condensates in neurodegeneration and cancer. Traffic. 2019; 20:890–911. 10.1111/tra.12704. 31606941

[6] Azaldegui CA, Vecchiarelli AG, Biteen JS The emergence of phase separation as an organizing principle in bacteria. Biophys J. 2021; 120:1123–38. 10.1016/j.bpj.2020.09.023. 33186556 PMC8059088

[7] Guo D, Xiong Y, Fu B et al. Liquid–liquid phase separation in bacteria. Microbiol Res. 2024; 281:127627 10.1016/j.micres.2024.127627. 38262205

[8] Harami GM, Kovacs ZJ, Pancsa R et al. Phase separation by ssDNA binding protein controlled via protein–protein and protein–DNA interactions. Proc Natl Acad Sci USA. 2020; 117:26206–17. 10.1073/pnas.2000761117. 33020264 PMC7584906

[9] Kozlov AG, Cheng X, Zhang H et al. How glutamate promotes liquid–liquid phase separation and DNA binding cooperativity of *E. coli* SSB protein. J Mol Biol. 2022; 434:167562 10.1016/j.jmb.2022.167562. 35351518 PMC9400470

[10] Zhao T, Liu Y, Wang Z et al. Super-resolution imaging reveals changes in *Escherichia coli* SSB localization in response to DNA damage. Genes Cells. 2019; 24:814–26. 31638317 10.1111/gtc.12729 PMC7065570

[11] Raghunathan S, Kozlov AG, Lohman TM et al. Structure of the DNA binding domain of *E. coli* SSB bound to ssDNA. Nat Struct Biol. 2000; 7:648–52. 10.1038/77943. 10932248

[12] Bonde NJ, Henry C, Wood EA et al. Interaction with the carboxy-terminal tip of SSB is critical for RecG function in *E. coli*. Nucleic Acids Res. 2023; 51:3735–53. 10.1093/nar/gkad162. 36912097 PMC10164576

[13] Dubiel K, Henry C, Spenkelink LM et al. Development of a single-stranded DNA-binding protein fluorescent fusion toolbox. Nucleic Acids Res. 2020; 48:6053–67. 10.1093/nar/gkaa320. 32374866 PMC7293020

[14] Antony E, Lohman TM Dynamics of *E. coli* single stranded DNA binding (SSB) protein–DNA complexes. Semin Cell Dev Biol. 2019; 86:102–11. 29588158 10.1016/j.semcdb.2018.03.017 PMC6165710

[15] Shereda RD, Reiter NJ, Butcher SE et al. Identification of the SSB binding site on *E. coli* RecQ reveals a conserved surface for binding SSB’s C terminus. J Mol Biol. 2009; 386:612–25. 10.1016/j.jmb.2008.12.065. 19150358 PMC2735845

[16] Shereda RD, Kozlov AG, Lohman TM et al. SSB as an organizer/mobilizer of genome maintenance complexes. Crit Rev Biochem Mol Biol. 2008; 43:289–318. 10.1080/10409230802341296. 18937104 PMC2583361

[17] Bonde NJ, Kozlov AG, Cox MM et al. Molecular insights into the prototypical single-stranded DNA-binding protein from *E. coli*. Crit Rev Biochem Mol Biol. 2024; 59:99–127. 10.1080/10409238.2024.2330372. 38770626 PMC11209772

[18] Kar M, Posey AE, Dar F et al. Glycine-rich peptides from FUS have an intrinsic ability to self-assemble into fibers and networked fibrils. Biochemistry. 2021; 60:3213–22. 10.1021/acs.biochem.1c00501. 34648275 PMC10715152

[19] Chong PA, Vernon RM, Forman-Kay JD RGG/RG motif regions in RNA binding and phase separation. J Mol Biol. 2018; 430:4650–65. 10.1016/j.jmb.2018.06.014. 29913160

[20] Murthy AC, Tang WS, Jovic N et al. Molecular interactions contributing to FUS SYGQ LC-RGG phase separation and co-partitioning with RNA polymerase II heptads. Nat Struct Mol Biol. 2021; 28:923–35. 34759379 10.1038/s41594-021-00677-4 PMC8654040

[21] Das S, Lin YH, Vernon RM et al. Comparative roles of charge, π, and hydrophobic interactions in sequence-dependent phase separation of intrinsically disordered proteins. Proc Natl Acad Sci USA. 2020; 117:28795–805. 10.1073/pnas.2008122117. 33139563 PMC7682375

[22] Harami GM, Pálinkás J, Kovács ZJ et al. Redox-dependent condensation and cytoplasmic granulation by human ssDNA binding protein 1 delineate roles in oxidative stress response. iScience. 2024; 27:110788. 39286502 10.1016/j.isci.2024.110788 PMC11403420

[23] Bianco PR, Stanenas AJ, Liu J et al. Fluorescent single-stranded DNA-binding proteins enable *in vitro* and *in vivo* studies. Methods Mol Biol. 2012; 922:235–44. 22976191 10.1007/978-1-62703-032-8_18 PMC5862424

[24] Waldman VM, Weiland E, Kozlov AG et al. Is a fully wrapped SSB–DNA complex essential for *Escherichia coli*survival?. Nucleic Acids Res. 2016; 44:4317–29. 10.1093/nar/gkw262. 27084941 PMC4872115

[25] Shereda RD, Bernstein DA, Keck JL A central role for SSB in *Escherichia coli* RecQ DNA helicase function. J Biol Chem. 2007; 282:19247–58. 10.1074/jbc.M608011200. 17483090

[26] Wang ZX An exact mathematical expression for describing competitive binding of two different ligands to a protein molecule. FEBS Lett. 1995; 360:111–4. 10.1016/0014-5793(95)00062-E. 7875313

[27] Sun J, Qu J, Zhao C et al. Precise prediction of phase-separation key residues by machine learning. Nat Commun. 2024; 15:2662 10.1038/s41467-024-46901-9. 38531854 PMC10965946

[28] Saar KL, Morgunov AS, Qi R et al. Learning the molecular grammar of protein condensates from sequence determinants and embeddings. Proc Natl Acad Sci USA. 2021; 118:e2019053118. 33827920 10.1073/pnas.2019053118 PMC8053968

[29] Chu X, Sun T, Li Q et al. Prediction of liquid–liquid phase separating proteins using machine learning. BMC Bioinformatics. 2022; 23:72 10.1186/s12859-022-04599-w. 35168563 PMC8845408

[30] Hardenberg M, Horvath A, Ambrus V et al. Widespread occurrence of the droplet state of proteins in the human proteome. Proc Natl Acad Sci USA. 2020; 117:33254–62. 10.1073/pnas.2007670117. 33318217 PMC7777240

[31] Orlando G, Raimondi D, Tabaro F et al. Computational identification of prion-like RNA-binding proteins that form liquid phase-separated condensates. Bioinformatics. 2019; 35:4617–23. 10.1093/bioinformatics/btz274. 30994888

[32] Vernon RM, Chong PA, Tsang B et al. Pi–pi contacts are an overlooked protein feature relevant to phase separation. eLife. 2018; 7:e31486 10.7554/eLife.31486. 29424691 PMC5847340

[33] Bolognesi B, Lorenzo Gotor N, Dhar R et al. A concentration-dependent liquid phase separation can cause toxicity upon increased protein expression. Cell Rep. 2016; 16:222–31. 10.1016/j.celrep.2016.05.076. 27320918 PMC4929146

[34] Wei MT, Elbaum-Garfinkle S, Holehouse AS et al. Phase behaviour of disordered proteins underlying low density and high permeability of liquid organelles. Nat Chem. 2017; 9:1118–25. 10.1038/nchem.2803. 29064502 PMC9719604

[35] Brady JP, Farber PJ, Sekhar A et al. Structural and hydrodynamic properties of an intrinsically disordered region of a germ cell-specific protein on phase separation. Proc Natl Acad Sci USA. 2017; 114:E8194–203. 28894006 10.1073/pnas.1706197114 PMC5625912

[36] Li HR, Chiang WC, Chou PC et al. TAR DNA-binding protein 43 (TDP-43) liquid–liquid phase separation is mediated by just a few aromatic residues. J Biol Chem. 2018; 293:6090–8. 10.1074/jbc.AC117.001037. 29511089 PMC5912450

[37] Lin Y, Currie SL, Rosen MK Intrinsically disordered sequences enable modulation of protein phase separation through distributed tyrosine motifs. J Biol Chem. 2017; 292:19110–20. 10.1074/jbc.M117.800466. 28924037 PMC5704491

[38] Tarakanova A, Huang W, Weiss AS et al. Computational smart polymer design based on elastin protein mutability. Biomaterials. 2017; 127:49–60. 10.1016/j.biomaterials.2017.01.041. 28279921 PMC9339145

[39] Zhao B, Li NK, Yingling YG et al. LCST behavior is manifested in a single molecule: elastin-like polypeptide (VPGVG)*_n_*. Biomacromolecules. 2016; 17:111–8. 10.1021/acs.biomac.5b01235. 26595324

[40] Rauscher S, Pomes R The liquid structure of elastin. eLife. 2017; 6:e26526 10.7554/eLife.26526. 29120326 PMC5703643

[41] Hong Y, Najafi S, Casey T et al. Hydrophobicity of arginine leads to reentrant liquid–liquid phase separation behaviors of arginine-rich proteins. Nat Commun. 2022; 13:7326 10.1038/s41467-022-35001-1. 36443315 PMC9705477

[42] Boeynaems S, De Decker M, Tompa P et al. Arginine-rich peptides can actively mediate liquid–liquid phase separation. Bio Protoc. 2017; 7:e2525 10.21769/BioProtoc.2525. PMC8413492 34541184

[43] Molliex A, Temirov J, Lee J et al. Phase separation by low complexity domains promotes stress granule assembly and drives pathological fibrillization. Cell. 2015; 163:123–33. 10.1016/j.cell.2015.09.015. 26406374 PMC5149108

[44] Bianco PR, Pottinger S, Tan HY et al. The IDL of *E. coli* SSB links ssDNA and protein binding by mediating protein–protein interactions. Protein Sci. 2017; 26:227–41. 10.1002/pro.3072. 28127816 PMC5275737

[45] Antony E, Weiland E, Yuan Q et al. Multiple C-terminal tails within a single *E. coli* SSB homotetramer coordinate DNA replication and repair. J Mol Biol. 2013; 425:4802–19. 10.1016/j.jmb.2013.08.021. 24021816 PMC3832242

[46] Najafi S, Lin Y, Longhini AP et al. Liquid–liquid phase separation of tau by self and complex coacervation. Protein Sci. 2021; 30:1393–407. 10.1002/pro.4101. 33955104 PMC8197434

[47] Collette D, Dunlap D, Finzi L Macromolecular crowding and DNA: bridging the gap between *in vitro* and *in vivo*. Int J Mol Sci. 2023; 24:17502 10.3390/ijms242417502. 38139331 PMC10744201

[48] Biswas S, Hecht AL, Noble SA et al. Understanding the impacts of molecular and macromolecular crowding agents on protein–polymer complex coacervates. Biomacromolecules. 2023; 24:4771–82. 10.1021/acs.biomac.3c00545. 37815312 PMC10646951

[49] Chao YC, Su SK, Lin YW et al. Interfacial properties of polyethylene glycol/vinyltriethoxysilane (PEG/VTES) copolymers and their application to stain resistance. J Surfactants Deterg. 2012; 15:299–305. 10.1007/s11743-011-1311-2. 22593640 PMC3338328

[50] Abramson J, Adler J, Dunger J et al. Accurate structure prediction of biomolecular interactions with AlphaFold 3. Nature. 2024; 630:493–500. 10.1038/s41586-024-07487-w. 38718835 PMC11168924

[51] Shishmarev D, Wang Y, Mason CE et al. Intramolecular binding mode of the C-terminus of *Escherichia coli* single-stranded DNA binding protein determined by nuclear magnetic resonance spectroscopy. Nucleic Acids Res. 2014; 42:2750–7. 10.1093/nar/gkt1238. 24288378 PMC3936761

[52] Su XC, Wang Y, Yagi H et al. Bound or free: interaction of the C-terminal domain of *Escherichia coli* single-stranded DNA-binding protein (SSB) with the tetrameric core of SSB. Biochemistry. 2014; 53:1925–34. 10.1021/bi5001867. 24606314

[53] Kerr ID, Wadsworth RI, Cubeddu L et al. Insights into ssDNA recognition by the OB fold from a structural and thermodynamic study of sulfolobus SSB protein. EMBO J. 2003; 22:2561–70. 10.1093/emboj/cdg272. 12773373 PMC156768

[54] Bianco PR The mechanism of action of the SSB interactome reveals it is the first OB-fold family of genome guardians in prokaryotes. Protein Sci. 2021; 30:1757–75. 10.1002/pro.4140. 34089559 PMC8376408

[55] Green M, Hatter L, Brookes E et al. Defining the intrinsically disordered C-terminal domain of SSB reveals DNA-mediated compaction. J Mol Biol. 2016; 428:357–64. 10.1016/j.jmb.2015.12.007. 26707201

[56] Curth U, Genschel J, Urbanke C et al. *In vitro* and *in vivo* function of the C-terminus of *Escherichia coli* single-stranded DNA binding protein. Nucleic Acids Res. 1996; 24:2706–11. 10.1093/nar/24.14.2706. 8759000 PMC145992

[57] Kozlov AG, Weiland E, Mittal A et al. Intrinsically disordered C-terminal tails of *E. coli* single-stranded DNA binding protein regulate cooperative binding to single-stranded DNA. J Mol Biol. 2015; 427:763–74. 10.1016/j.jmb.2014.12.020. 25562210 PMC4419694

[58] Sandler SJ, Bonde NJ, Wood EA et al. The intrinsically disordered linker in the single-stranded DNA-binding protein influences DNA replication restart and recombination pathways in *Escherichia coli* K-12. J Bacteriol. 2024; 206:e0033023 10.1128/jb.00330-23. 38470036 PMC11025327

[59] Feliciello I, Ljubic S, Dermic E et al. Single-strand DNA-binding protein suppresses illegitimate recombination in *Escherichia coli*, acting in synergy with RecQ helicase. Sci Rep. 2024; 14:20476 10.1038/s41598-024-70817-5. 39227621 PMC11372144

[60] Pancsa R, Tompa P Essential functions linked with structural disorder in organisms of minimal genome. Biol Direct. 2016; 11:45 10.1186/s13062-016-0149-y. 27608806 PMC5016991

[61] Grigorev V, Wingreen NS, Zhang Y Conformational entropy of intrinsically disordered proteins bars intruders from biomolecular condensates. PRX Life. 2025; 3:013011 10.1103/PRXLife.3.013011. PMC13246162 42266718

[62] Wang B, Zhang L, Dai T et al. Liquid–liquid phase separation in human health and diseases. Signal Transduct Target Ther. 2021; 6:290 10.1038/s41392-021-00678-1. 34334791 PMC8326283

[63] Pakravan D, Orlando G, Bercier V et al. Role and therapeutic potential of liquid–liquid phase separation in amyotrophic lateral sclerosis. J Mol Cell Biol. 2021; 13:15–28. 10.1093/jmcb/mjaa049. 32976566 PMC8036000

