# Supplementary material for: Selective engineering of condensation properties of single-stranded DNA binding (SSB) protein via its intrinsically disordered linker region
Source: Nucleic Acids Res. 2025 Jun 6;53(11):gkaf481. doi: 10.1093/nar/gkaf481 (PMC12143593; doi:10.1093/nar/gkaf481)
Supplement: gkaf481_Supplemental_File [file gkaf481_supplemental_file.docx]

**SUPPLEMENTARY INFORMATION**


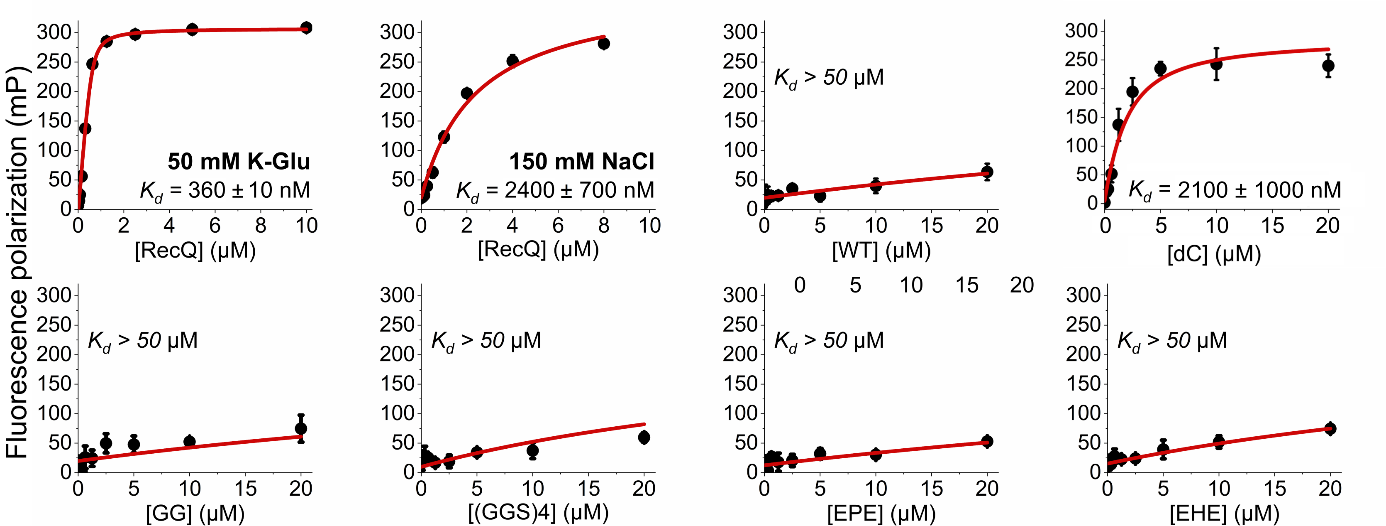


**Fig. S1. Interaction of the isolated SSB C-terminal peptide (CTP) with RecQ helicase and SSB constructs**

Results of FP experiments are shown in which 25 nM flCTP was titrated with RecQ helicase (first panel: 50 mM K-Glu as used in ([1](#_ENREF_1)), second panel: 150 mM NaCl as used in ([2](#_ENREF_2))), WT SSB, dC and SSB variants in which the IDL is drastically changed (GG, (GGS)4, EPE and EHE). Data points are the mean ± SD of three independent experiments. Lines show best fits based on a quadratic binding equation (see Methods). *K*_d_ values are shown in each plot. The results show that the isolated flCTP can bind to RecQ helicase and the OB domains of dC tetramers. However, the same peptide is unable to effectively compete with the CTP segments harbored in the WT, GG, (GGS)4, EPE and EHE SSB tetramers.


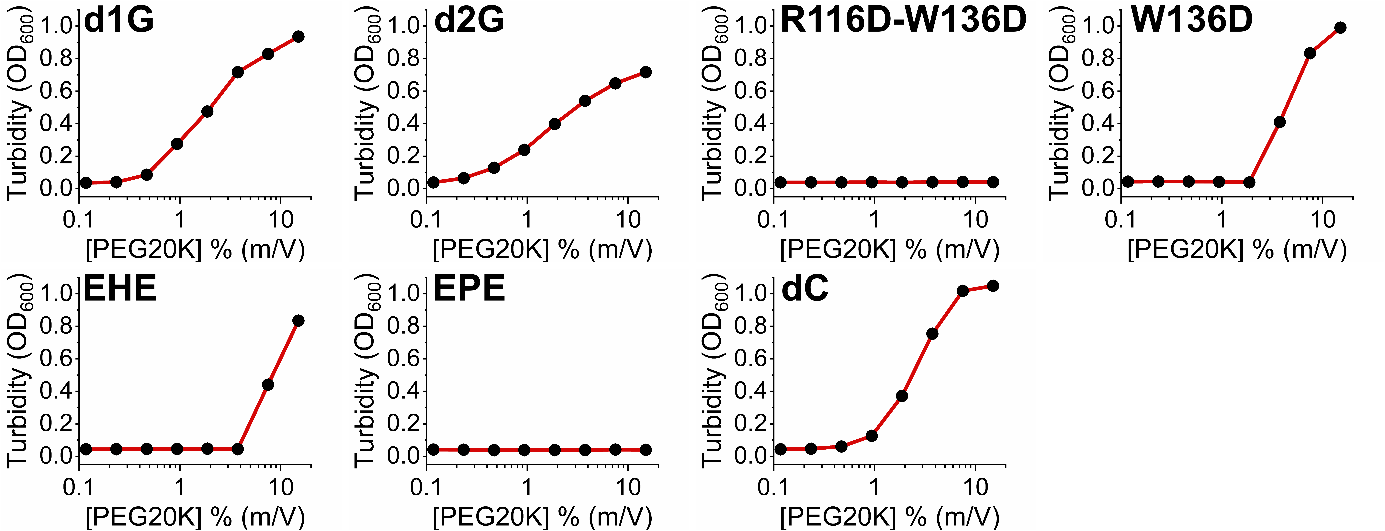


**Fig. S2. Molecular crowder (PEG 20K) concentration dependence of condensate formation by EcSSB constructs**

Turbidity (*OD*_600_) values of samples of EcSSB variants (20 µM) are shown. Data points represent the mean ± SD of three independent experiments. Error bars are within symbols.


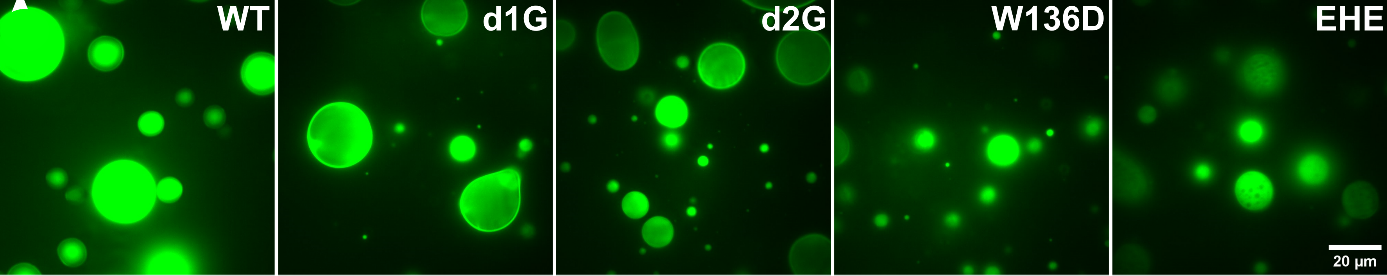


**Fig. S3. Epifluorescence images of condensates formed by EcSSB constructs**

Condensates of EcSSB constructs (20 µM, as indicated in the panels) imaged in the presence of 10% (m/V) PEG 20K molecular crowder. Condensates were visualized using 0.3 µM fluorescently labeled WT EcSSB. Images were taken after mixing.

**Table S1. Interaction of EcSSB variants with ssDNA, RecQ helicase and intermolecular OB domains**

|  | **ssDNA_36_** | | | | **dT_79_** | | | **RecQ** | | **Intermolecular OB** | |
| --- | --- | --- | --- | --- | --- | --- | --- | --- | --- | --- | --- |
|  | **K_d_ ± SD (nM)** | ***p*-value (N=3)** | | **n**  **(Stoichiometry)** | **K_d_ ± SD (nM)** | ***p*-value (N=3)** | **n**  **(Stoichiometry)** | **K_d_ ± SD (nM)** | ***p*-value (N=3)** | **K_d_ ± SD (nM)** | ***p*-value (N=3)** |
| **WT** | 32 ± 15 | |  | 1.9 ± 0.1 | < 10 |  | 1.7 ± 0.3 | < 10 |  | 2500 ± 600 |  |
| **d1G** | 89 ± 36 | 0.29 | | 2.3 ± 0.3 | 11 ± 2.6 | 0.18 | 2.2 ± 0.6 | < 10 | 0.16 | 220 ± 90 | 0.04 |
| **d2G** | 46 ± 8.0 | 0.43 | | 2.8 ± 0.3 | 11 ± 2.5 | 0.21 | 1.8 ± 0.6 | 11 ± 3.0 | 0.33 | 110 ± 80 | 0.04 |
| **(GGS)4** | 16 ± 5.9 | 0.27 | | 1.9 ± 0.3 | < 10 | 0.99 | 2.2 ± 0.6 | 17 ± 4.0 | 0.17 | 19 ± 16 | 0.002 |
| **GG** | 12 ± 6.2 | 0.28 | | 2.4 ± 0.2 | < 10 | 0.20 | 2.3 ± 0.7 | ־ | ־ | 97 ± 26 | 0.004 |
| **R116D-W136D** | 21 ± 2.3 | 0.35 | | 2 ± 0.1 | < 10 | 0.99 | 1.8 ± 0.6 | 17 ± 2.0 | 0.60 | 2500 ± 400 | 0.72 |
| **W136D** | 16 ± 4.3 | 0.15 | | 2 ± 0.3 | < 10 | 0.19 | 1.7 ± 0.5 | 29 ± 5.0 | 0.10 | ־ | ־ |
| **EPE** | 84 ± 19 | 0.75 | | 2.2 ± 0.2 | < 10 | 0.41 | 1.8 ± 0.6 | < 10 | 0.25 | 57 ± 35 | 0.008 |
| **EHE** | 96 ± 26 | 0.51 | | 2.1 ± 0.4 | < 10 | 0.37 | 2.3 ± 1 | < 10 | 0.20 | 2300 ± 400 | 0.77 |

Dissociation constants (*K*_d_) were determined in FP assays (**Figs. 2, 5**). *K*_d_ values refer to the interactions of EcSSB variants with ssDNA (ssDNA_36_ or dT_79_), RecQ helicase or intermolecular OB domains. Values reported are mean ± SD of the best-fit parameters of three independent measurements. Pairwise Student`s t-tests were carried out for values of WT *versus* each variant. Note that *K*_d_ values convey significant fitting error due to applied fluorescent reporter concentrations of 10 nM (ssDNA oligonucleotides) and 25 nM (flCTP). Stoichiometry was defined as mol SSB tetramer per mol oligonucleotide molecule.

**Table S2. Half-maximal effective concentrations to disrupt EcSSB condensates**

|  | **dT_79_ (µM)** | **NaCl (mM)** | **K-Glu (mM)** | **L-Arg (mM)** |
| --- | --- | --- | --- | --- |
| **WT** | 4.5 ± 0.1 | 52 ± 3 | >500 | 51 ± 5 |
| **WT*** | 4.8 ± 0.4 | 97 ± 1 | >500 | 55 ± 13 |
| **d1G*** | 3.7 ± 0.3 | 30 ± 2 | >500 | 18 ± 2 |
| **d2G*** | 5.5 ± 0.7 | 35 ± 3 | 130 ± 6 | 19 ± 1 |
| **W136D*** | 3.1 ± 0.2 | 6 ± 1 | 20 ± 5 | 7 ± 3 |
| **dC*** | 5.7 ± 1.3 | 27 ± 4 | 28 ± 3 | 11 ± 2 |

Values were determined in turbidity assays (**Fig. 6**) and show the mean ± SD of the best-fit parameters of three independent measurements. Quadratic equation ([1](#_ENREF_1)) was used in case of dT_79_ and Hill equation in case of others. Asterisks (*) indicate samples assessed in the presence of 3 % (m/V) PEG 20K molecular crowder.

**Table S3. Properties of AlphaFold 3 predicted structures of EcSSB constructs**

| **Structure ID** | **OB site occupied by CTP ^a^** | **CTP binding configuration ^b^** | **OB residues in contact with the last four residues of CTP (DIPF) ^c^** | **Helix within IDL ^d^** |
| --- | --- | --- | --- | --- |
| WT #0 | S1 | Trans (A-CD) | Chain C: N26, F61, R85, **V106**; Chain D: **W41**, D43, **K44**, A45, K50 | N |
| WT #1 | S1 | Trans (A-ACD) | Chain A: G107; Chain C: N105, **V106**, G107; Chain D: W41, R42, D43, K44 | N |
| WT #2 | S2 | Trans (A-CD) | Chain C: **T86**, K88, **Y98**, T100; Chain D: **T34, W55**, H56, R57, T100 | N |
| WT #3 | -- | -- | -- | N |
| WT #4 | S1 | Cis (A-AB) | Chain A: M1, V106; Chain B: W41, **K44** | N |
| GG #0 | S3 | Cis/Trans (A-ACD) | Chain A: **K63,** V67, **Y71**; Chain C: A2; Chain D: M49 | N |
| GG #1 | S3 | Cis/Trans (A-AC) | Chain A: **K63**, V67, Y71; Chain C: M1, A2 | N |
| GG #2 | S3 | Cis/Trans (A-ACD) | Chain A: **K63**, Y71; Chain C: A2; Chain D: M49 | N |
| GG #3 | S3 | Cis/Trans (A-ACD) | Chain A: **K63,** V67, Y71; Chain C: A2; Chain D: M49 | N |
| GG #4 | S3 | Cis/Trans (A-AC) | Chain A: **K63**, V67, Y71, ; Chain C: M1, A2 | N |
| (GGS)4 #0 | S2 | Trans (A-CD) | Chain C: T86, **Y98**; Chain D: G16, Q17, **T34, W55,** H56, R57 | N |
| (GGS)4 #1 | S2 | Trans (A-CD) | Chain C: T86, Y98; Chain D: G16, Q17, **T34, W55**, H56, **R57** | N |
| (GGS)4 #2 | S2 | Trans (A-CD) | Chain C: T86, **Y98**; Chain D: G16, Q17, **T34, W55,** H56, R57 | N |
| (GGS)4 #3 | S2 | Trans (A-CD) | Chain C: T86, **Y98**, T100; Chain D: G16, Q17, **T34, W55,** H56, R57 | N |
| (GGS)4 #4 | S2 | Trans (A-CD) | Chain C: T86, **Y98**; Chain D: G16, Q17, **T34, W55,** H56, R57 | N |
| EHE #0 | S2 | Cis (A-AB) | Chain A: Q17, **T34**, W55, **R57,** T99, E101; Chain B: T86, K88, D96, **Y98** | Y (P129-T133) |
| EHE #1 | S2 | Cis (A-AB) | Chain A: Q17, T34, **R57;** Chain B: **Y98** | Y (P129-S132) |
| EHE #2 | S1 | Cis (A-AB) | Chain A: **M1**, M24, N26, F61, **R85**, **N105,** V106; Chain B: E39, W41, K50, **Q52** | Y (P129-S132) |
| EHE #3 | S1 | Cis (A-AB) | Chain A: M1, M24, N26, **F61**, **N105,** V106; Chain B: **W41**, D43, K44, K50, Q52 | N |
| EHE #4 | S2 | Cis (A-A) | Chain A: E20, **R22**, **N32**, R57, **R87**, W89, T90, D91, Q92, E101 | N |
| EPE #0 | S2 | Cis (A-AB) | Chain A: **Y98**; Chain B: Q17, T34, W55, **R57** | Y (D116-N146) |
| EPE #1 | S2 | Cis (A-AB) | Chain A: Q17, T34, **W55**, R57; Chain B: T86, Y98 | Y (D116-N145) |
| EPE #2 | S1 | Cis (A-A) | Chain A: V106 | Y (D116-N146) |
| EPE #3 | S1 | Cis (A-AB) | Chain A: M1, M24, F61, R85, **N105,** V106; Chain B: **W41**, D43, K44, K50 | Y (D116-G150) |
| EPE #4 | S2 | Cis (A-AB) | Chain A: Q17, **T34**, E54, **W55**, H56, **R57**; Chain B: T86, Y98 | Y (K117-N144) |
| d1G #0 | S1 | Cis (A-AB) | Chain A: M1, N26, F61, V106; Chain B: **K44** | N |
| d1G #1 | -- | -- | -- | N |
| d1G #2 | S1 | Cis (A-AB) | Chain A: **M1**, M24, F61, R85, **N105, V106**; Chain B: **W41**, D43, K50, Q52 | N |
| d1G #3 | -- | -- | -- | N |
| d1G #4 | S1 | Cis (A-AB) | Chain A: **M1**, M24, N26, **F61**, **R85, N105, V106**; Chain B: E39, **W41**, D43, K44, K50, Q52 | N |
| d2G #0 | S1 | Trans (A-CD) | Chain C: **W41**, D43, K44, A45, K50; Chain D: M1, M24, M26, **F61**, G62, R85, **V106** | N |
| d2G #1 | S1 | Trans (A-CD) | Chain C: **W41**, D43, K44, K50; Chain D: **M1**, M24, F61, R85, V106 | N |
| d2G #2 | S1 | Trans (A-CD) | Chain C: **W41**, D43, K44, K50; Chain D: M1, M24, N26, F61, R85, **N105,** **V106** | N |
| d2G #3 | S2 | Trans (A-C) | Chain C: R87, K88 | N |
| d2G #4 | -- | .. | -- | N |
| W136D #0 | S1 | Trans (A-CD) | Chain C: **W41**, D43, **K44**, K50; Chain D: M1, M24, N26, **F61**, G62, R85, N105, **V106** | N |
| W136D #1 | S1 | Trans (A-CD) | Chain C: **W41**, D43, K44, K50, Q52, Chain D: **M1**, M24, **F61,** R85, V106 | N |
| W136D #2 | S1 | Trans (A-CD) | Chain C: **W41**, D43, K44, K50; Chain D: M1, M24, N26, F61, R85, V106 | N |
| W136D #3 | -- | - | - | N |
| W136D #4 | S1 | Cis/Trans (A-ABC) | Chain A: **M1**, V106; Chain B: R42; Chain C: **K63**, L64, N105, V106, G108, T109 | N |
| R116D-W136D #0 | -- | - | - | N |
| R116D-W136D #1 | S2 | Trans (A-D) | Chain D: Q17, E20, R22, N32, T34, **R57, R87,** W89, E101 | N |
| R116D-W136D #2 | -- | - | - | N |
| R116D-W136D #3 | S1 | Cis (A-AB) | Chain A: M1, M24, N26, **F61**, R85, N105, **V106**; Chain B: E39, **W41**, D43, K44, K50 | N |
| R116D-W136D #4 | S2 | Cis (A-AB) | Chain A: Q17, **W55**, R57; Chain B: Y98 | N |
| dC #0 | -- | -- | -- | N |
| dC #1 | -- | -- | -- | N |
| dC #2 | -- | -- | -- | N |
| dC #3 | -- | -- | -- | N |
| dC #4 | -- | -- | -- | N |
| Δ120-166 #0 | S2 | Trans (A-CD) | Chain C: T86, **Y98**; Chain D: G16, Q17, **T34**, **W55**, H56, R57 | N |
| Δ120-166 #1 | S2 | Trans (A-CD) | Chain C: **Y98**; Chain D: G16, Q17, **T34**, **W55**, H56, R57 | N |
| Δ120-166 #2 | S2 | Trans (A-CD) | Chain C: T86, **Y98**; Chain D: G16, Q17, **T34**, **W55**, H56, R57 | N |
| Δ120-166 #3 | S2 | Trans (A-CD) | Chain C: T86, **Y98**; Chain D: G16, Q17, **T34**, **W55**, H56, R57 | N |
| Δ120-166 #4 | S2 | Trans (A-CD) | Chain C: T86, **Y98**; Chain D: G16, Q17, **T34**, **W55**, H56, R57 | N |
| Δ130-166 #0 | S2 | Trans (A-CD) | Chain C: T86, **Y98**; Chain D: G16, **T34**, **W55**, H56, **R57** | N |
| Δ130-166 #1 | S2 | Trans (A-CD) | Chain C: T86, **Y98**; Chain D: G16, Q17, **T34**, **W55**, H56, R57 | N |
| Δ130-166 #2 | S2 | Trans (A-CD) | Chain C: T86, **Y98**; Chain D: G16, Q17, **T34**, **W55**, H56, R57 | N |
| Δ130-166 #3 | S2 | Trans (A-CD) | Chain C: T86, **Y98**; Chain D: G16, Q17, **T34**, **W55**, H56, R57 | N |
| Δ130-166 #4 | S2 | Trans (A-CD) | Chain C: **Y98**; Chain D: G16, Q17, **T34**, **W55**, H56, R57 | N |
| Δ151-166 #0 | S1 | Trans (A-CD) | Chain C: **W41**, D43, K44, K50; Chain D: M24, N26, F61, R85, **N105, V106** | N |
| Δ151-166 #1 | S1 | Trans (A-CD) | Chain C: **W41**, D43, K44, K50; Chain D: **M1**, M24, N26, F61, R85, **N105, V106** | N |
| Δ151-166 #2 | -- | -- | -- | N |
| Δ151-166 #3 | -- | -- | -- | N |
| Δ151-166 #4 | -- | -- | -- | N |

^a^ See Discussion for nomenclature of CTP binding sites within the OB domain.

^b^ ’Cis’ denotation means that the CTP binds within the same OB dimer (CTP of chain A within dimer AB), while ‘Trans’ denotation means that the CTP binds to the other OB dimer (composed of chains C and D). Models were classified based on the majority of CTP-contacting residues. ‘Cis/Trans’ denotation was used when several CTP contacting residues were provided by both OB dimers.

^c^ Listed are chain IDs and residues that lie within 5 Å distance from any of the last four residues of the CTP of chain A (sequence DIPF). Distances of all heavy atoms were considered. Residues contacted by at least two residues of the specified CTP segment are highlighted in bold.

^d^ Helix start and end residues are indicated for chain A. Slight (1-2 aa) variations at both ends of the helices were observed among the four IDLs in some of the structures. N means no helix.

Among the AF3 models of IDL-engineered EcSSB variants, those of GG showed a clear preference toward CTP binding into a unique site on the interface of the two OB dimers (S3, blue in **Fig. 9 C**). The short IDL of GG does not allow the CTP to reach either S1 or S2, which could contribute to this construct’s inability to undergo LLPS due to reduced conformational dynamics and interaction multivalency. Moreover, aggregation of GG (**Fig. 3**) is probably a result of the loss of the shielding function of the IDL, leading to association of aggregation-prone EcSSB tetramers. The lack of or reduced LLPS of variants with short but glycine-rich IDLs ((GGS)4 here in **Fig. 3** and constructs of ∆120-166, ∆130-166 in another work ([3](#_ENREF_3))) could be linked to all of them being restricted to adopting S2 in Trans configuration. This restricted OB-CTP binding pattern probably stems from increased local concentrations of CTPs around S2 due to the shortened IDL and, in turn, to reduced dynamics and multivalency, which is unfavorable for LLPS.

For the chimeric EPE variant, AF3 models unanimously showed the formation of a 30-residue α-helix in the N-terminal part of *P. falciparum* IDL (between residues D116-N146, with some variation). In the AF2-predicted monomeric structure of PfSSB a shorter helix is present (residues K196-E211 in PfSSB, equivalent to residues K117-E132 in EPE). The single available experimental PfSSB structure (UniProt: Q8I415, PDB: 3ULP) ([4](#_ENREF_4)) contains only the first few residues of the IDL; thus, the long α-helix identified here by AF3 is a new indication. Nevertheless, its formation agrees with the hydrodynamic properties and simulation results reported by Kozlov *et al.* ([5](#_ENREF_5)) suggesting that the PfSSB IDL samples more expanded conformations than does EcSSB IDL. They also concluded that it is the IDL, and not the DNA binding core, of PfSSB that prevents formation of the (SSB)_35_ ssDNA binding mode. Therefore, this unique functional property of PfSSB could well be linked to the presence of the long α-helix detected here. Interestingly, three of the five AF3 models of EHE also suggest a small α-helix of only one turn (between residues P129-S132) in the N-terminal part of the IDL, which interacts with the OB tetramer surface in two models. The lack of and reduced LLPS of the EPE and EHE variants, respectively (**Fig. 3**), could be linked to the long and very stable or short and transient α-helices observed at the beginning of their respective IDLs, as such secondary structure formation likely restricts the conformational dynamics of the IDL. Accordingly, the binding preference of these two variants is restricted to bound CTP states in Cis configuration, mostly to S2.

Interestingly, the d1G and d2G variants lacking one or two N-terminal G-rich motifs in their IDLs both showed clear preference toward S1-bound and unbound CTP states according to AF3. However, d1G prefers binding in Cis configuration, while d2G exclusively binds in Trans. Again, these restricted preferences imply reduced multivalency. Due to changes in charge patterning within its IDL, the W136D variant probably samples a more expanded conformation compared to WT EcSSB, as also evidenced by its slower migration in SDS-PAGE (**Fig. 1**). According to AF3 models, W136D can sample unbound CTP states, but CTP binding seems to be restricted to S1 that could provide an explanation for its reduced LLPS propensity. Nevertheless, AF3 models provided no clear structural explanation for the loss of LLPS propensity in R116D-W136D. R116 lies at the very beginning of the IDL within the first glycine-rich motif at which the IDLs of two neighboring monomers are still in relative proximity. Charge repulsion between the two IDL stems could contribute to keeping them apart, but in principle, the native R in both IDLs could lead to similar charge repulsion as D. Accordingly, AF3 models with unbound CTPs of WT and R116D-W136D appeared very similar. Therefore, we propose that R116D and W136D probably affect IDL dynamics (introduce a shift towards more expanded conformations as evidenced by SDS-PAGE (**Fig. 1**) and may convert potential intra- or intertetramer IDL-IDL cation-π interactions (which are invisible for AF3) to charge repulsion.

AF3 models of the Δ151-166 variant showed a shift toward unbound CTP states and a restriction to S1 in Trans configuration in the bound states. Interestingly, although the N-terminal part of the IDL remained unchanged, in two of three models with unbound CTP a relatively long segment of the N-terminal part of the IDL interacted with the OB tetramer in a way not seen for other variants. It is unclear how the removal of the C-terminal part affects the behavior of the N-terminal IDL segment and how it could lead to increased condensation propensity compared to WT, as implied by the data of Kozlov *et al.* ([3](#_ENREF_3)). We note that the precise nature of phase-separated particles formed by the C-terminal IDL deleted mutants, including Δ151-166, is unknown as their condensation propensity was monitored solely by turbidimetry, which is unsuitable for precise distinction between liquid particles and *e.g.* amorphous aggregates. Nevertheless, AF3 models of the variants discussed above clearly imply that the properties of the N-terminal IDL segment largely impact the intramolecular binding preferences of the CTP, which in turn also impact LLPS propensity.

**Table S4. Comparison of experimentally determined and predicted LLPS propensities of EcSSB constructs**

| **Construct** | **WT** | **GG** | **(GGS)4** | **EHE** | **EPE** | **d1G** | **d2G** | **W136D** | **R116D-W136D** | **dC** | **Δ120-166 (ref Kozlov 2022)** | **Δ130-166**  **(ref Kozlov 2022)** | **Δ151-166**  **(ref Kozlov 2022)** |
| --- | --- | --- | --- | --- | --- | --- | --- | --- | --- | --- | --- | --- | --- |
| **Experimen-tal LLPS** | **Yes** | **No** | **No** | **Stro-ngly redu-ced** | **No** | **Redu-ced** | **Redu-ced** | **Redu-ced** | **No** | **Redu-ced** | **Redu-ced** | **Increa-sed** | **Increa-sed** |
| **PSPHunter** | 0.795 (Y) | 0.181 (N) | 0.390 (N) | 0.698 (Y) | 0.582 (Y) | 0.715 (Y) | 0.563 (Y) | 0.806 (Y) | 0.803 (Y) | 0.799 (Y) | 0.220 (N) | 0.352 (N) | 0.700 (Y) |
| **DeePhase** | 0.60  (Y) | 0.072  (N) | 0.10  (N) | 0.70  (Y) | 0.60  (Y) | 0.56  (Y) | 0.55  (Y) | 0.59  (Y) | 0.60  (Y) | 0.6  (Y) | 0.24  (N) | 0.30  (N) | 0.52  (Y) |
| **PSPredictor** | 0.730 (Y) | 0.021 (N) | 0.089 (N) | 0.542 (Y) | 0.213 (N) | 0.333 (N) | 0.058 (N) | 0.582 (Y) | 0.543 (Y) | 0.769 (Y) | 0.046 (N) | 0.022 (N) | 0.203 (N) |
| **FuzDrop** | 0.868 (Y) | 0.315 (N) | 0.465 (N) | 0.965 (Y) | 0.668 (Y) | 0.770 (Y) | 0.628 (Y) | 0.823 (Y) | 0.909 (Y) | 0.744 (Y) | 0.429 (N) | 0.406 (N) | 0.66(Y) |
| **Pscore** | 5.60 (Y) | (TS) | (TS) | 3.72 (N) | -1.17 (N) | 3.99 (N) | 1.92 (N) | 5.73 (Y) | 5.98  (Y) | 5.50 (Y) | (TS) | 0.35  (N) | 5.26  (Y) |
| **PSPer** | 0.306 (N) | - | 0.195 (N) | 0.292 (N) | 0.303 (N) | 0.285 (N) | 0.272 (N) | 0.309 (N) | 0.307 (N) | 0.304 (N) | 0.192 (N) | 0.224 (N) | 0.274 (N) |
| **CatGranule** | 1.885 (Y) | 0.595 (Y) | 1.405 (Y) | 0.810 (Y) | 1.342 (Y) | 1.391 (Y) | 0.921 (Y) | 1.931 (Y) | 1.936 (Y) | 1.962 (Y) | 0.834 (Y) | 1.397 (Y) | 2.019 (Y) |

LLPS propensities predicted by seven available *in silico* methods are shown for EcSSB IDL-engineered variants assessed in the current study and in ref. ([3](#_ENREF_3)). Values were rounded to three decimal digits in cases where the outputs were provided with more than three digits. Yes (Y) or No (N) indicate binary evaluation of predicted LLPS propensities. PSPHunter, PSPredictor, FuzDrop and DeePhase are 2^nd^-generation general predictors using a 0-1 scale wherein a value above 0.5 indicates LLPS propensity. PScore is a 1^st^-generation predictor based on pi-pi intrachain interresidue interactions. It uses a unique scale wherein a value above 4 indicates that the protein can drive LLPS. It has a minimum sequence length requirement of 140 residues; thus, constructs GG, (GGS)4, and Δ120-166 were too short (TS) to evaluate using this method. PSPer is a 1^st^-generation predictor trained on FUS-family RNA binding proteins that has a 0-1 scale wherein a value above 0.5 indicates LLPS propensity. CatGranule is a 1^st^-generation method trained on granule-localized yeast proteins. It predicts rather a propensity for localization to membraneless organelles (MLOs) than a propensity to drive LLPS, with a stringent lower threshold of 0.5.

While PSPer that was only trained on FUS-family RNA binding proteins did not even predict WT EcSSB to be LLPS-prone, nor any of the IDL-engineered variants, CatGranule that was trained on a set of yeast proteins localizing to ribonucleoprotein (RNP) granules (mostly client proteins) predicted WT EcSSB as well as all tested variants as LLPS-prone. PScore showed the best performance among first-generation methods as it could identify WT EcSSB as LLPS-prone and correctly predicted loss of LLPS propensity for several variants with large IDL deletions or substitutions (EHE, EPE, d1G, d2G), but still failed to identify the reduced LLPS propensity of variants with limited modifications (dC, W136D, R116D-W136D). The relatively good performance of PScore could imply that π-π interactions significantly contribute to EcSSB LLPS, since this method was specifically trained to evaluate LLPS based on such interactions ([6](#_ENREF_6)). Notably, none of the methods could specifically predict the reduction/loss in LLPS propensity for the W136D and R116D-W136D variants. Interestingly, all predictors gave a similar LLPS propensity score to dC as to WT, but most of them correctly assigned a markedly reduced LLPS score to d1G, even though these two variants represent a similar reduction in IDL length (8 *versus* 7 residues, respectively). This finding implies that predictors are generally more sensitive to changes affecting glycine-rich regions. Furthermore, while Kozlov *et al.* ([3](#_ENREF_3)) reported an increased LLPS propensity for two IDL-truncated variants (Δ130-166 and Δ150-166), none of the predictors could correctly identify this unexpected tendency (most predicted the opposite), probably because the lengths of IDRs are usually positively correlated with LLPS propensity, and the methods are currently unable to recognize exceptions to this rule.

**References**

1. Harami, G.M., Kovacs, Z.J., Pancsa, R., Palinkas, J., Barath, V., Tarnok, K., Malnasi-Csizmadia, A. and Kovacs, M. (2020) Phase separation by ssDNA binding protein controlled via protein-protein and protein-DNA interactions. *Proceedings of the National Academy of Sciences of the United States of America*, **117**, 26206-26217.

2. Shereda, R.D., Bernstein, D.A. and Keck, J.L. (2007) A central role for SSB in Escherichia coli RecQ DNA helicase function. *The Journal of biological chemistry*, **282**, 19247-19258.

3. Kozlov, A.G., Cheng, X., Zhang, H., Shinn, M.K., Weiland, E., Nguyen, B., Shkel, I.A., Zytkiewicz, E., Finkelstein, I.J., Record, M.T., Jr. *et al.* (2022) How Glutamate Promotes Liquid-liquid Phase Separation and DNA Binding Cooperativity of E. coli SSB Protein. *Journal of molecular biology*, **434**, 167562.

4. Antony, E., Weiland, E.A., Korolev, S. and Lohman, T.M. (2012) Plasmodium falciparum SSB tetramer wraps single-stranded DNA with similar topology but opposite polarity to E. coli SSB. *Journal of molecular biology*, **420**, 269-283.

5. Kozlov, A.G., Weiland, E., Mittal, A., Waldman, V., Antony, E., Fazio, N., Pappu, R.V. and Lohman, T.M. (2015) Intrinsically disordered C-terminal tails of E. coli single-stranded DNA binding protein regulate cooperative binding to single-stranded DNA. *Journal of molecular biology*, **427**, 763-774.

6. Vernon, R.M., Chong, P.A., Tsang, B., Kim, T.H., Bah, A., Farber, P., Lin, H. and Forman-Kay, J.D. (2018) Pi-Pi contacts are an overlooked protein feature relevant to phase separation. *eLife*, **7**, e31486
